# Two chloroplast thioredoxin systems differentially modulate photosynthesis in Arabidopsis depending on light intensity and leaf age

**DOI:** 10.1111/tpj.14959

**Published:** 2020-08-31

**Authors:** Manuel Guinea Diaz, Lauri Nikkanen, Kristiina Himanen, Jouni Toivola, Eevi Rintamäki

**Affiliations:** ^1^ Molecular Plant Biology Department of Biochemistry University of Turku Turku 20520 Finland; ^2^ National Plant Phenotyping Infrastructure University of Helsinki Helsinki 00790 Finland

**Keywords:** Calvin cycle enzymes, thylakoid electron transfer, chloroplast redox state

## Abstract

Various regulatory mechanisms have evolved in plants to optimize photosynthetic activity under fluctuating light. Thioredoxins (TRX) are members of the regulatory network balancing activities of light and carbon fixation reactions in chloroplasts. We have studied the impact of two chloroplast TRX systems, the ferredoxin‐dependent TRX reductase (FTR) and the NADPH‐dependent TRX reductase C (NTRC) on regulation of photosynthesis by mutants lacking or overexpressing a component of either system. Plants were subjected to image‐based phenotyping and chlorophyll fluorescence measurements that allow long‐term monitoring of the development and photosynthetic activity of the rosettes, respectively. Our experiments demonstrate that NTRC and FTR systems respond differently to variation of light intensity. NTRC was an indispensable regulator of photosynthesis in young leaves, at light‐intensity transitions and under low light intensities limiting photosynthesis, whereas steady‐state exposure of plants to growth or higher light intensities diminished the need of NTRC in regulation of photosynthesis. In fluctuating light, overexpression of NTRC increased the quantum yield of Photosystem II (YII) at low light and stimulated the relaxation of non‐photochemical quenching (NPQ) after high light exposure, indicating that overexpression of NTRC improves leaf capacity to convert light energy to chemical energy under these conditions. Overexpression of chimeric protein (NTR‐TRXf) containing both the thioredoxin reductase and TRXf activity on an *ntrc* mutant background, did not completely recover either growth or steady‐state photosynthetic activity, whereas OE‐NTR‐TRXf plants exposed to fluctuating light regained the wild‐type level of Y(II) and NPQ.

## Significance Statement

Plants have evolved numerous mechanisms for regulation of photosynthesis under natural fluctuating light, but this strict regulation limits photosynthetic efficiency. We demonstrate that the NADPH‐dependent TRX reductase C (NTRC) balances the activities of photosynthetic light and carbon fixation reactions at transitions between light intensities and overexpression of NTRC improves leaf capacity to convert light energy to chemical energy by moderating light‐intensity dependent regulation of photosynthesis and resulting in an increase in leaf photosynthesis.

## INTRODUCTION

Photosynthesis comprises a series of highly regulated reactions that convert solar energy and CO_2_ to chemical energy of carbohydrates in chloroplasts. The need for strong regulation derives from the dangerous nature of light and the variation in its quality and quantity, which exposes the photosynthetic machinery to photodamage and induces generation of reactive oxygen species (ROS) under ever‐changing environments (Roach and Krieger‐Liszkay, 2014; Tikkanen and Aro, 2014; Mattila *et al*., 2015). Optimization of photosynthesis under fluctuating light conditions requires strict balancing of the reactions absorbing light energy with the reactions fixing carbon into photosynthetic end products. Photosynthetic light harvesting and energy conversion is regulated by multiphase network, including non‐photochemical quenching (NPQ) of light energy in Photosystem II (PSII) antenna, regulation of linear and cyclic electron flow (CEF) and generation of proton motive force (*pmf*) in thylakoid membranes, regulation of the enzymes in carbon metabolism in stroma, as well as the control of generation and scavenging of ROS in chloroplasts (Tikkanen and Aro, 2014; Goldschmidt‐Clermont and Bassi, 2015; Dietz *et al*., 2016; Ruban, 2016; Yamori and Shikanai, 2016; Armbruster *et al*., 2017). Recently, chloroplast thioredoxins (TRX) have been shown to play a vital role in this regulatory network (Thormählen *et al*., 2017; Pérez‐Ruiz *et al*., 2017; Nikkanen *et al*., 2018, 2019; Vaseghi *et al*., 2018). TRXs are oxidoreductases that modify the function of cellular proteins by cleaving disulphide bridges in redox‐active domains of proteins. Thioredoxin reductases (TR) mediate metabolic and environmental signals to TRXs. Two TRX systems exist in plant chloroplasts. The ferredoxin‐TRX (Fd‐TRX) system is activated by light via reduction of Fd, and consists of the Fd‐dependent thioredoxin reductase (FTR) and f‐, m‐, y‐, x‐ and z‐type TRXs (Buchanan, 2016; Geigenberger *et al*., 2017). The other system is NADPH‐dependent TR C (NTRC), a protein containing both TR and TRX domains within one polypeptide (Serrato *et al*., 2004). It takes electrons from NADPH and can be active both in illuminated and dark‐adapted leaves (Pérez‐Ruiz *et al*., 2006; Nikkanen *et al*., 2018).

**Figure 1:**
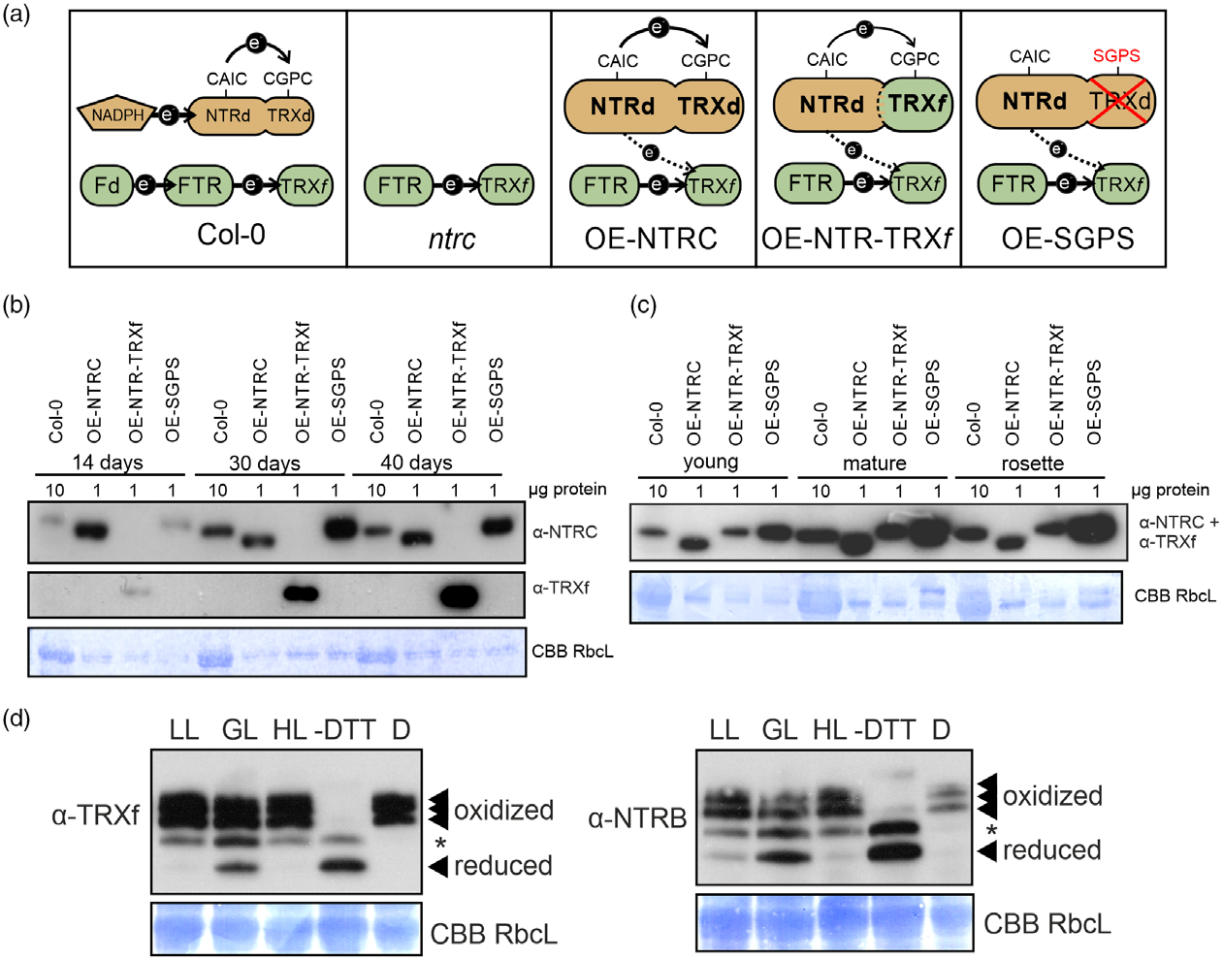
Accumulation of overexpressed proteins in transgenic Arabidopsis lines. (a) Schematic illustration of the relative content and electron transfer reactions of NADPH‐dependent TRX reductase C (NTRC) and ferredoxin‐dependent TRX reductase (FTR) systems in transgenic lines. (b, c) Content of NTRC, NTRC_SGPS_ and NTR‐TRXf proteins in rosettes of different age. Soluble proteins were extracted from the whole rosettes (b) or separately from young or mature leaves of 50 days old plants (c). Proteins were separated in sodium dodecyl sulphate–polyacrylamide gel electrophoresis before Western blotting with anti‐NTRC or anti‐TRXf1/f2 antibody. Amount of loaded soluble protein (µg) is indicated above a blot. Coomassie‐stained band of Rubisco was used as loading control. (d) Relative amount of reduced and oxidized forms of a chimeric NTR‐TRXf protein in dark‐adapted leaves (D) or in leaves illuminated for 2 h at 50 (low light, LL), 200 (growth light, GL) and 800 (high light, HL) μmol photons m^−2^ sec^−1^. Total leaf proteins were extracted in the presence of 10% trichloroacetic acid to preserve the thiol redox state and labelled with methoxypolyethylene glycol maleimide (MAL‐PEG) as described in Experimental procedures. Proteins were separated in sodium dodecyl sulphate–polyacrylamide gel electrophoresis before Western blotting with anti‐TRXf1/f2 and anti‐NTRB antibodies. –DTT indicates the negative control sample not treated with DTT before MAL‐PEG incubation. *Unknown conformation of NTR‐TRXf protein.

Initially the Fd‐TRX system was assigned to activate photosynthetic reactions in dark‐to‐light transitions (Buchanan, 2016), whereas NTRC was shown to be an important scavenger of ROS by transferring reducing equivalents from NADPH to chloroplast 2‐Cys peroxiredoxins (2‐Cys Prx) (Pérez‐Ruiz *et al*., 2006; Pulido *et al*., 2010). Today, a number of publications have revealed that a high number of chloroplast proteins and processes are targeted to TRX regulation (Geigenberger *et al*., 2017; Cejudo *et al*., 2019; Nikkanen and Rintamäki, 2019). Characterization of *Arabidopsis thaliana* mutants combining deficiencies in both systems, such as mutants devoid of NTRC and TRXf (Thormählen *et al*., 2015; Ojeda *et al*., 2017), NTRC and TRXm (Da *et al*., 2018), NTRC and TRXx (Ojeda *et al*., 2017), or NTRC and the catalytic subunit of FTR (Yoshida and Hisabori, 2016), provided strong evidence that both TRX systems act concertedly in regulation of photosynthesis. In addition to carbon and antioxidant metabolisms TRX systems regulate also the light reactions in thylakoid membranes (Rintamäki *et al*., 2000; Hisabori *et al*., 2013; Courteille *et al*., 2013; Carrillo *et al*., 2016; Naranjo *et al*., 2016b; Nikkanen *et al*., 2018, 2019). This co‐regulation has been suggested to keep the electron transfer reactions and the photosynthetic carbon metabolism in balance to avoid photodamage of the photosynthetic machinery under fluctuating light conditions (Thormählen *et al*., 2017; Nikkanen *et al*., 2018). Recently, a model of chloroplast redox regulation was proposed suggesting that the redox balance of 2‐Cys PRXs exerts a key role in maintaining the thiol redox homeostasis of the chloroplast (Pérez‐Ruiz *et al*., 2017; Vaseghi *et al*., 2018). However, it is still unclear how the regulatory tasks of photosynthesis are coordinated between Fd‐TRX and NTRC systems and how the systems respond to variation in light intensity. To address the questions we have constructed transgenic Arabidopsis TRX lines, where the amount or the activity of either the NTRC or the Fd‐TRX system, or both is altered (Figure 1). The transgenic lines were analysed using the National Plant Phenotyping Infrastructure at Helsinki University (NaPPI) (https://www.helsinki.fi/en/infrastructures/national‐plant‐phenotyping) that allows determination of the photosynthetic activity of the lines during the entire plant life cycle using non‐invasive techniques (Figure S1). The results support our discovery that overexpression of NTRC (OE‐NTRC) stimulates the growth of Arabidopsis (Toivola *et al*., 2013; Nikkanen *et al*., 2016). We show that the increased content of NTRC improves the quantum yield of PSII and diminishes the dissipation of light energy as heat in Arabidopsis leaves at growth (GL) and lower light (LL) intensities. Improved function of PSII was particularly prominent under fluctuating light in plants overexpressing NTRC. However, in the absence of NTRC an increase in the content of TRXf, representing the Fd‐TRX system, was sufficient to recover normal function of photosynthesis under fluctuating light.

### RESULTS

### Amount of NTRC, NTRC_SGPS_ and chimeric NTR‐TRXf in transgenic rosettes at different ages

To investigate the specific roles of NTRC and TRXf in the regulation of chloroplast processes we constructed transgenic lines on an *ntrc* background overexpressing wild‐type NTRC protein (OE‐NTRC line), NTRC protein with an active NTR domain but an inactive TRX domain (NTRC_SGPS_) (OE‐SGPS line), and a line with a chimeric protein, where the *TRXf* sequence was fused with the sequence of the *NTR* domain of NTRC (OE‐NTR‐TRXf line). We chose to overexpress the chimeric protein instead of the *NTR* domain and *TRXf* separately, as we have earlier shown that overexpression of the *NTR* domain without the TRX domain resulted in anomalous folding of NTR protein in Arabidopsis (Toivola *et al*., 2013). Wild‐type Columbia‐0 (*Col‐0*), *ntrc*, OE‐NTRC and OE‐SGPS lines have endogenous TRXf content, while all overexpression lines have the higher content of a functional NTR domain of NTRC (Figure 1a). In addition, OE‐NTRC has extra TRX activity of NTRC and OE‐NTR‐TRXf extra TRXf protein in the chloroplast (Figure 1a).

The amount of NTRC from soluble leaf proteins was relatively stable in OE‐NTRC rosettes of different ages, while increased amounts of NTRC_SGPS_, and NTR‐TRXf proteins accumulated in transgenic leaves during the growth of rosettes, with young leaves containing significantly less overexpressed protein than mature leaves (Figure 1b,c). Next, we analysed if the overexpressed TRXs carry out reduction/reoxidation cycle *in vivo* such as endogenous chloroplast TRXs. We have previously demonstrated that the extra NTRC in OE‐NTRC plants is reducible and that the amount of fully reduced NTRC is fairly constant in OE‐NTRC, both in dark‐adapted plants and in illuminated leaves under various light intensities (Nikkanen *et al*., 2018). Furthermore, the amount of the reduced form of endogenous TRXf is increased in OE‐NTRC leaves (Nikkanen *et al*., 2016). The redox state of the NTRC reductase domain in NTRC_SGPS_ depends on light intensity (Nikkanen *et al*., 2018), indicating that NTRC_SGPS_ is active *in vivo*. We have also shown that OE‐NTRC_SGPS_ increases the amount of reduced TRXf in LL‐illuminated leaves (Nikkanen *et al*., 2016), suggesting that NTRC_SGPS_ is able to transfer electrons to TRXf. In this study, we have estimated the relative amount of the reduced form of the chimeric NTR‐TRXf in leaves of the OE‐NTR‐TRXf line (Figure 1d) by using both a TRXf antibody and an NADPH‐dependent TR B (NTRB) antibody recognizing the NTR domain of NTRC (Toivola *et al*., 2013). The highest amount of reduced NTR‐TRXf protein was present at GL, whereas oxidized forms were dominant in dark‐adapted, LL‐ and high light (HL) illuminated leaves. This means that all overexpressed NTRC forms are functional *in vivo* in illuminated leaves (Figure 1a).

### Growth capacity of the transgenic lines with modified chloroplast TRX systems

**Figure 2:**
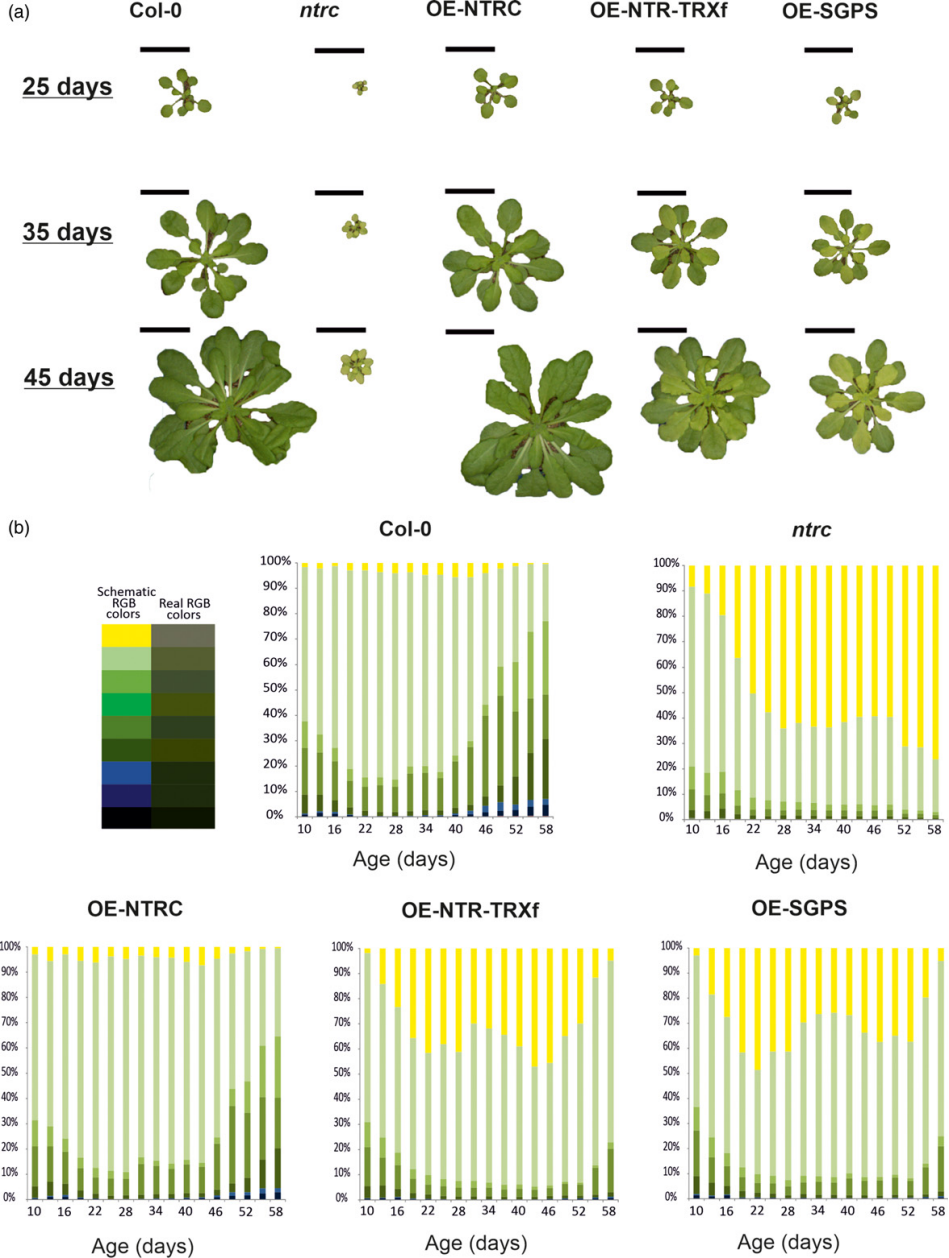
Development and pigmentation of Arabidopsis rosettes. (a) Rosettes of Arabidopsis wild‐type (*Col‐0*), *NTRC* knockout mutant (*ntrc*) and transgenic lines overexpressing (OE) modified TRX systems (OE‐NTRC, OE‐NTR‐TRXf, OE‐SGPS) were grown in short day conditions (8 h light/16 h dark) at 200 μmol photons m^−2^ sec^−1^ and the photos were taken at ages of 25, 35 and 45 days after stratification. Scale bar is 4 cm. (b) Plants were imaged daily by an overhead CCD camera for RGB images. Percentage of different colours in the rosette was calculated (Awlia *et al*., 2016) and the values at every third day are presented in the figure. Colour code indicates the correspondence between recorded colours of the rosette (Real RGB colours) and the colours presented in the figure (Schematic RGB colours).

To follow the pigment content in leaves we quantified the changes in rosette colour (see Experimental procedures). The higher proportion of dark green colour in ageing rosettes indicates increased leaf chlorophyll content (Figure 2). The yellow colour that dominates the colour profile of *ntrc* (Figure 2b) derives from poor development and the reduced number of chloroplasts in mesophyll cells (Lepistö *et al*., 2009; Lepistö and Rintamäki, 2012; Toivola *et al*., 2013).

No major differences in pigmentation were detected in 10‐day‐old seedlings (Figure 2b) consisting mainly of cotyledons (Figure S2) because the lack of active NTRC has no significant effect on pigmentation of cotyledons. However, the coloration of transgenic plants lacking fully functional NTRC changed remarkably in comparison with *Col‐0* and OE‐NTRC, when the first true leaves developed (Figure 2a,b). The decrease in green colour was largest in *ntrc*, while the proportion of pale green leaves first increased in young rosettes of OE‐SGPS and OE‐NTR‐TRXf and then diminished during the expansion of leaves (Figure 2b), probably due to the accumulation of NTRC_SGPS_ and chimeric NTR‐TRXf in leaves (Figure 1c). The second greening of OE‐SGPS and OE‐NTRC‐TRXf leaves occurred in ageing rosettes, but they never reached the colour pattern of *Col‐0* and OE‐NTRC rosettes.

Rosette growth correlated with the changes in colour patterns (Figure 2a,b). No large difference was seen in cotyledon sizes between the lines (Figure S2), whereas the growth of young true leaves was clearly retarded in plants lacking fully functional NTRC (Figure 2a). In comparison with *ntrc*, the size and number of leaves partially recovered in OE‐SGPS, and even more in OE‐NTR‐TRXf (Figure 2a), indicating that the chimeric NTRC‐TRXf protein is active in the chloroplast and it compensated the lack of NTRC better than the NTRC_SGPS_ form. The experiment indicated that proper development of chloroplasts is strongly dependent on NTRC and only an extra amount of TRXf can compensate for the absence of NTRC.

### Photosynthetic characterization of transgenic rosettes

**Figure 3:**
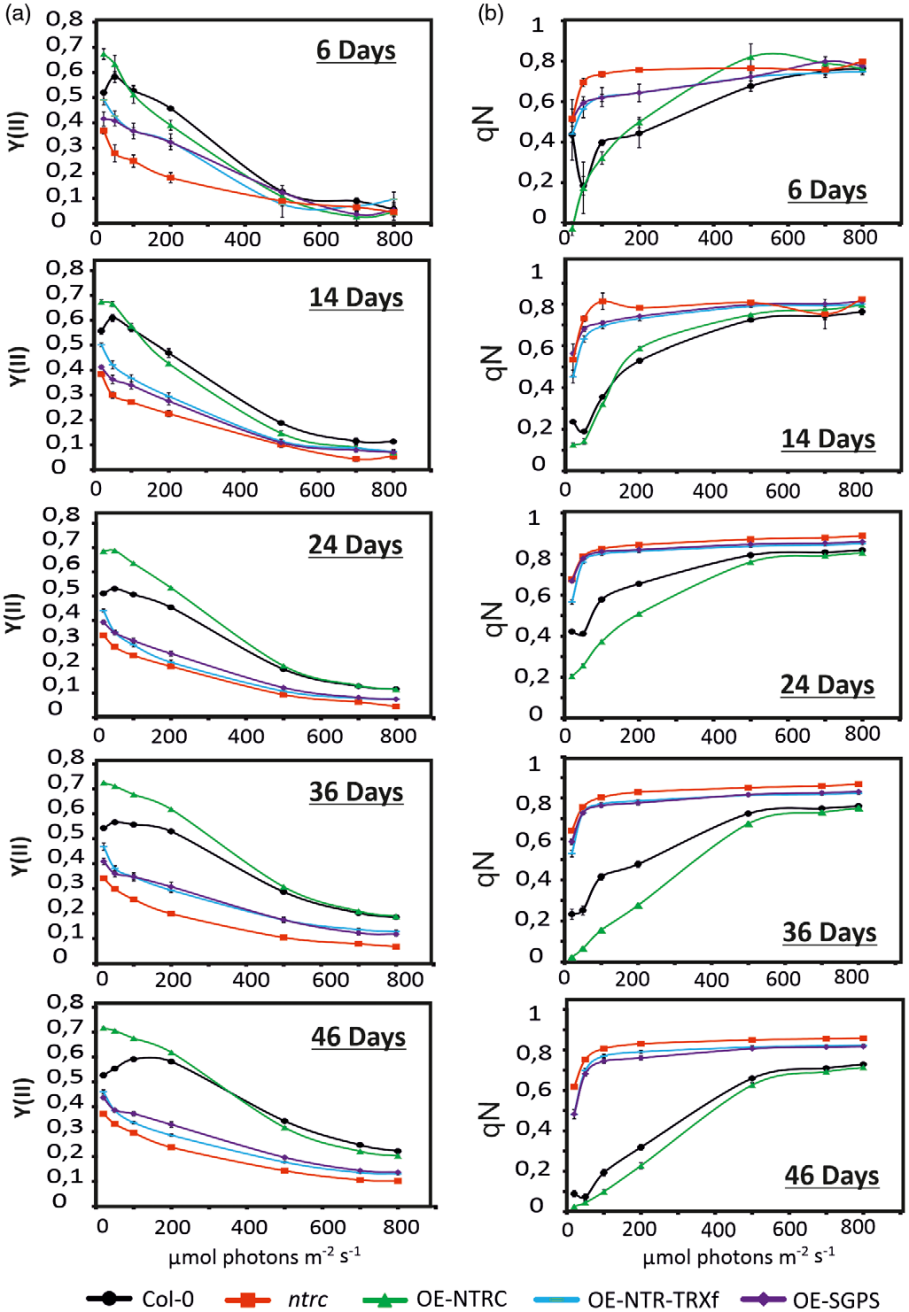
Light curve of quantum yield of Photosystem II (Y(II)) and non‐photochemical quenching (NPQ; qN) determined for rosettes at different ages. (a, b) Plants of *Col‐0* (black dots), *ntrc* (red squares) and transgenic lines overexpressing (OE) modified TRX systems [OE‐NTRC (green triangles), OE‐NTR‐TRXf (cyan lines), OE‐SGPS (purple diamonds)] were grown in short day conditions (8 h light/16 h dark) at 200 photons μmol m^−2^ sec^−1^. Chl*a* fluorescence of the whole rosette at different ages was measured by applying the light curve protocol symbol, and used to calculate the quantum yield of Y(II) (a) and NPQ (qN) (b). Values are means of 15–20 plants of each line ± SEM. SEM bars are only seen if the bar is higher than the symbol.

The photosynthetic characterization of the transgenic lines was carried out at NaPPi by using non‐invasive chlorophyll *a* (Chl*a*) fluorescence techniques (Figure S1). The parameters presented in Figures 3 and 5 are derived from the fluorescence recorded from whole rosettes.

The recovery of growth capacity of OE‐NTRC, OE‐SGPS and OE‐NTR‐TRXf lines correlated with the restoration of the quantum yield of PSII (YII) in illuminated rosettes (Figure 3a). In young seedlings of OE‐NTRC, Y(II) was slightly higher than in *Col‐0* only at very low light intensity, whereas at the age of ≥24 days the capability of PSII to utilize light energy was significantly higher in OE‐NTRC rosettes than in *Col‐0* under GL or LL (Figure 3a). Among the transgenic lines, *ntrc* showed the lowest Y(II) under every light condition and at all ages tested, with the differences between *ntrc* and other lines being more pronounced under LL and GL (Figure 3a). A slightly higher Y(II) was recorded for OE‐SGPS and OE‐NTR‐TRXf rosettes than in *ntrc* under LL and GL intensities. This suggests that TRXf can poorly compensate for the lack of TRX activity of NTRC in OE‐SGPS and OE‐NTR‐TRXf leaves.

The light curve of NPQ (qN) indicated that, as for Y(II), qN of OE‐NTRC seedlings did not substantially differ from *Col‐0* (Figure 3b), whereas after the seedling stage, OE‐NTRC rosettes dissipated significantly less light energy as heat than *Col‐0* at LL and GL (Figure 3b). Accordingly, in the transgenic lines lacking active TRXd of NTRC, NPQ was strongly induced at LL. This indicates that an increase in the activity or the amount of TRXf was not sufficient to restore the wild‐type response of NPQ to light intensity in the absence of the TRX domain of NTRC.

### Redox state of photosynthetic proteins in transgenic lines

Next, we estimated the redox states of redox‐regulated enzymes representing the light reactions (γ subunit of ATP synthase, CF1γ), CBB cycle (phosphoribulokinase, PRK), and antioxidant activity in the chloroplast (2‐Cys PRXs) of 35‐day‐old plants showing clear differences in photosynthetic parameters between the lines (Figure 3). 2‐Cys PRXs are reported to be primarily reduced by NTRC (Pérez‐Ruiz *et al*., 2006; Pulido *et al*., 2010; Nikkanen *et al*., 2016), and CF1γ and PRK both by NTRC and TRXf (Brandes *et al*., 1996; Schwarz *et al*., 1997; Nikkanen *et al*., 2016; Carrillo *et al*., 2016).

**Figure 4:**
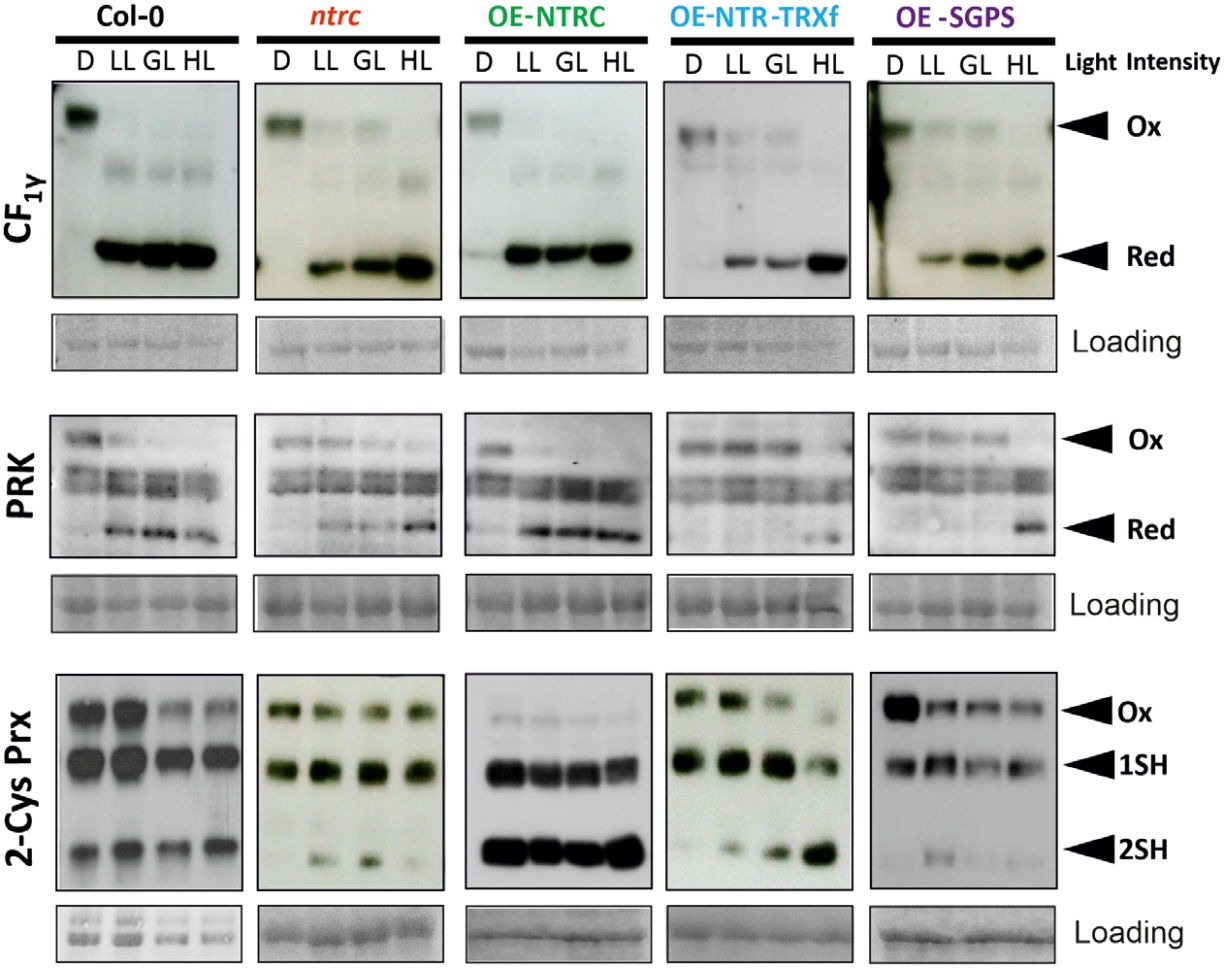
Redox states of the γ‐subunit of ATP synthase (CF1γ), phosphoribulokinase (PRK) and 2‐Cys peroxiredoxins (2‐Cys PRX) in dark‐adapted plants and plants illuminated under various irradiances. Leaves from 35‐day‐old *Col‐0*, *ntrc* and transgenic lines overexpressing (OE) modified TRX systems (OE‐NTRC, OE‐NTR‐TRXf, and OE‐SGPS) were collected at the end of the night (D), or from plants illuminated for 2 h at 50 (low light, LL), 200 (growth light, GL) and 800 (high light, HL) μmol photons m^−2^ sec^−1^ and frozen in liquid nitrogen. Total leaf proteins were extracted and separated in sodium dodecyl sulphate–polyacrylamide gel electrophoresis as described in Figure 1 before Western blotting with anti‐2‐Cys Prx antibody, anti‐Cf_1γ_ antibody and anti‐PRK antibody. Bands corresponding to the oxidized (Ox) and reduced (Red) form of the proteins are marked in the figure. 2‐Cys PRX exist as three redox forms, fully oxidized (Ox), partially reduced (1SH) and fully reduced (2SH) (Nikkanen *et al*., 2016). Ponceau staining was used as the loading control.

A proportion of the ATP synthase pool was already activated by reduction of CF1γ in dark‐adapted leaves of OE‐NTRC and full reduction of CF1γ was detected both in *Col‐0* and OE‐NTRC leaves under all light intensities tested (Figure 4). Reduction of CF1γ was impaired in all transgenic lines lacking an active TRX domain of NTRC under illumination at LL and GL (Figure 4). To study the activation of ATP synthase at dark‐to‐light transitions further we monitored the decay of the electrochromic shift (ECS) signal in fully expanded dark‐adapted leaves, representing the activity of ATP synthase in darkness (Figure S3a–e) and determined the proton conductivity of the thylakoid membrane (gH+) (Cruz *et al*., 2005) at dark‐to‐LL transitions (Figure S3f). The decay of the ECS signal was faster in dark‐adapted OE‐NTRC leaves and slower in *ntrc* than in *Col‐0*, whereas only minor differences were observed in OE‐SGPS and OE‐NTR‐TRXf leaves in comparison with *Col‐0*. Proton conductivity of thylakoid membrane during the first seconds of illumination correlated with the decay of flashed‐induced ECS signal in dark‐adapted leaves, showing that gH+ in OE‐NTRC was substantially higher than in any other line. Moreover, the gH+ value remained low in *ntrc*, whereas it increased in other lines illuminated for 1 min (Figure S3f), suggesting that the enhancement of TRXf activity in OE‐SGPS and OE‐NTRC‐TRXf rescued the ability to raise gH+ when illumination continued.

A trend similar to the redox regulation of CF1γ was also observed for PRK in transgenic lines lacking NTRC with a substantial amount of reduced PRK form only in leaves exposed to HL (Figure 4). Contrary to other studied lines, reduced PRK was the dominant form of the enzyme in illuminated OE‐NTRC leaves and a proportion of reduced PRK was present even in dark‐adapted leaves. Likewise, only fully reduced and half‐oxidized forms of 2‐Cys PRXs were present both in dark‐adapted and illuminated leaves of OE‐NTRC, whereas half and fully oxidized 2‐Cys PRXs were dominant in transgenic lines lacking NTRC (Figure 4). However, OE‐NTR‐TRXf leaves illuminated at HL contained a substantial amount of fully reduced 2‐Cys PRXs, suggesting that chimeric NTR‐TRXf was able to transmit electrons to 2‐Cys PRXs under these conditions.

### Photosynthetic response of transgenic lines to light fluctuation

**Figure 5:**
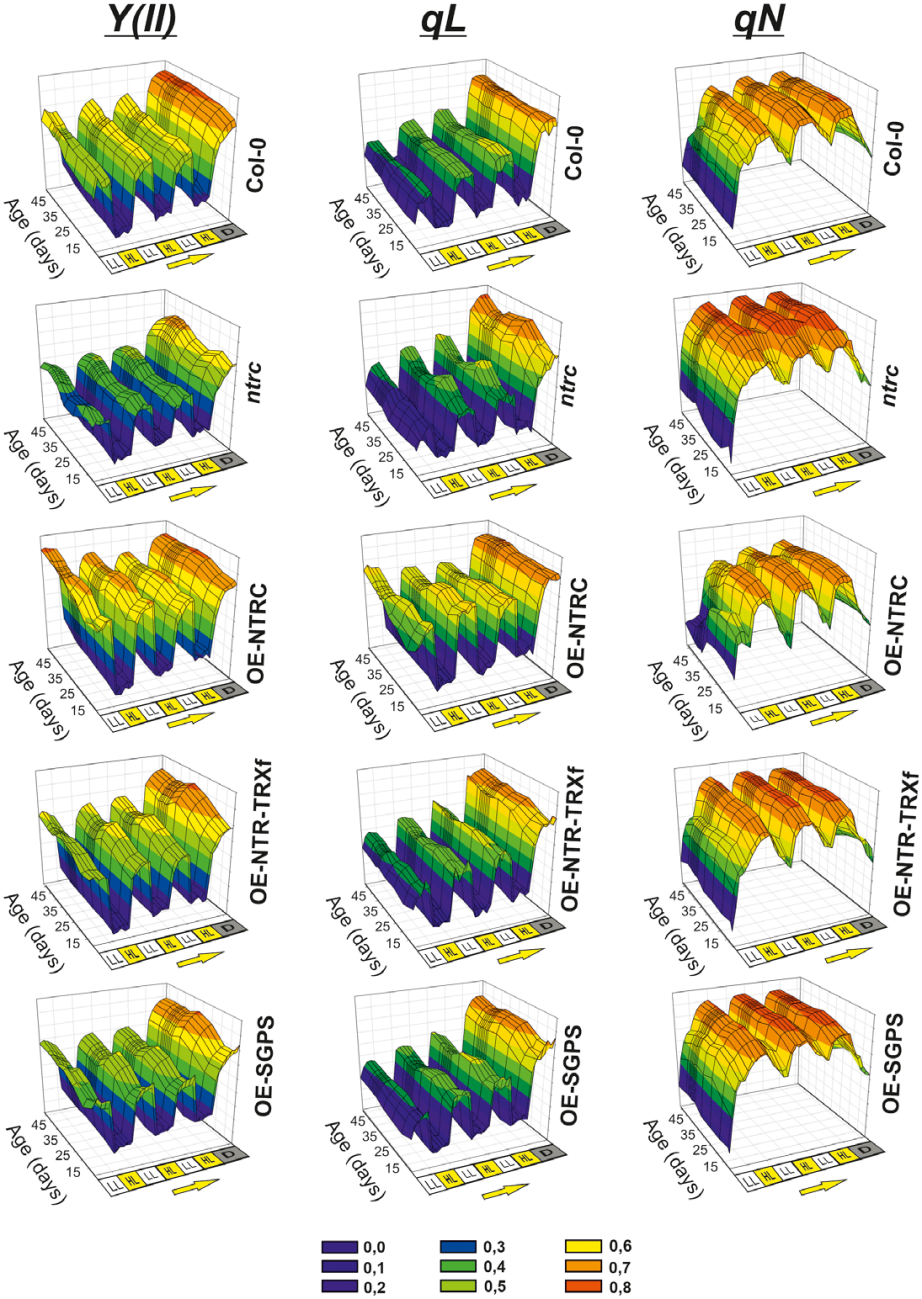
Photosynthetic characterization of transgenic lines under fluctuating light. Rosettes of *Col‐0, ntrc*, and transgenic lines overexpressing (OE) modified TRX systems (OE‐NTRC, OE‐NTR‐TRXf, and OE‐SGPS) were dark‐adapted for 20 min and then exposed to three low light/high light (LL: 50 μmol photons m^−2^ sec^−1^; HL: 800 μmol photons m^−2^ sec^−1^) cycles. Chlorophyll *a* fluorescence was measured to calculate the Photosystem II quantum yield (YII), photochemical quenching (qL) and non‐photochemical quenching (qN) of rosettes. Mean value of 15–20 plants of each line is presented in the figure from day 11 until day 52 after stratification. Yellow arrow indicates the direction of the light transition according to quenching protocol. Colour codes of the scale are indicated below the figure.

**Figure 6:**
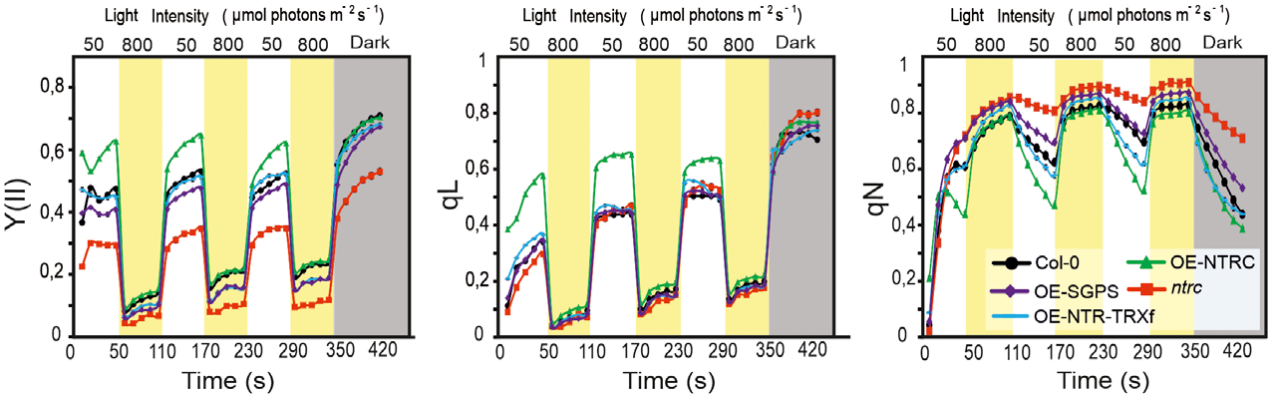
Photosynthetic parameters in 25‐day‐old transgenic lines exposed to fluctuating light. Rosettes of *Col‐0* (black dots), *ntrc* (red squares) and transgenic lines overexpressing (OE) modified TRX systems [OE‐NTRC (green triangles), OE‐NTR‐TRXf (cyan lines), OE‐SGPS (purple diamonds)] were exposed to low light/high light cycles as described in Figure 5. Chl*a* fluorescence was monitored during the treatments of plants and used for calculation of Photosystem II quantum yield (YII), photochemical quenching (qL) and non‐photochemical quenching (qN). Mean values of 15–20 plants ± SEM are presented in the figure. SEM bars are only seen if the bar is higher than the symbol.

It has recently been proposed that chloroplast TRXs, particularly NTRC, are involved in the regulation of chloroplast redox balance under fluctuating light conditions (Geigenberger *et al*., 2017; Thormählen *et al*., 2017; Nikkanen *et al*., 2018). To investigate the role of NTRC and TRXf under repetitive light fluctuations, the transgenic lines were exposed to three LL/HL cycles once per week in the first 4 weeks of growth and daily in the fifth week of growth (Figure 5). A quenching protocol (Figure S1c) was used to estimate Y(II), photochemical quenching (qL) and NPQ (qN) in rosettes. A quenching experiment of 25‐day‐old plants is presented in Figure 6.

Major differences in photosynthetic performance between the transgenic lines were observed at the LL phases of fluctuating light (Figures 5 and 6). The experiment shows that OE‐NTRC was superior to *Col‐0* and other lines in maintaining and recovering active photosynthesis (YII, qL) at dark‐to‐light transition and during LL periods after HL treatments, respectively. Both parameters were significantly higher in OE‐NTRC than in any other lines indicating improved utilization of light energy at LL phases of light treatment (Figures 5 and 6). The qL parameter describes the proportion of open PSII centres in leaves (Kramer *et al*., 2004). Thus, high qL combined with raised quantum yield of PSII suggests elevated activity of PSI acceptor side reactions in OE‐NTRC chloroplasts. Accordingly, faster relaxation of NPQ at LL after HL treatments was recorded for OE‐NTRC rosettes (Figure 6). This ability to maintain higher photosynthetic activity at LL phases of fluctuating light endures in OE‐NTRC rosettes from seedlings to 7‐week‐old rosettes (Figure 5). Some fluctuations of the photosynthetic parameters were observed at different ages of both *Col‐0* and OE‐NTRC but the differences in photosynthetic performance between OE‐NTRC and *Col‐0* remained similar throughout the growth of plants.

In *ntrc,* Y(II) was low both under constant (Figure 3) and fluctuating light (Figures 5 and 6). Strongest defects in Y(II) were detected under LL (Figures 3 and 6), thus supporting the hypothesis that NTRC has the highest impact on photosynthesis under irradiances limiting photosynthesis. The low Y(II) in *ntrc* was associated with high NPQ (Figures 3, 5 and 6). Hardly any relaxation of NPQ occurred in *ntrc* at LL phases after HL treatments, particularly in young rosettes (Figures 5 and 6). The qL value recorded at LL phases of the treatment fluctuated throughout the growth of *ntrc* (between 0.4 and 0.5), peaking at young and old rosettes (Figure 5). In young *ntrc* rosettes, qL was only slightly lower in comparison with *Col‐0* (Figure 6). This increase of qL was associated with a simultaneous rise of NPQ at LL phases of fluctuating light (Figures 5 and 6), indicating that a substantial proportion of the light energy absorbed by PSII was dissipated as heat in the antennae and consequently less energy was transferred to the PSII reaction centre, which is observed as low Y(II).

In comparison with *ntrc*, photosynthetic performance at fluctuating light was partially recovered in OE‐SGPS. At LL phases of fluctuating light Y(II) and qL were close to the level of *Col‐0*. At dark‐to‐LL transitions NPQ induction resembled that in *ntrc,* but after the first HL phase the relaxation of NPQ at LL was significantly faster than in *ntrc* (Figures 5 and 6). This suggests that a short HL treatment of OE‐SGPS rosettes activates process(es) that promote(s) NPQ relaxation.

Although steady‐state photosynthetic performance was only partially recovered by overexpression of the chimeric NTR‐TRXf protein on an *ntrc* background (Figure 3), it restored Y(II), qL and qN to *Col‐0*‐levels during dark‐to‐light transitions and in rapidly fluctuating light (Figures 5 and 6). Relaxation of NPQ at LL after HL treatment was even faster than in *Col‐0*, but never as fast as in OE‐NTRC. This suggests that TRXf can compensate for the absence of endogenous NTRC under fluctuating light, but only when the TRXf activity is substantially higher than in *Col‐0* chloroplasts. The dose effect was also seen in the relaxation of NPQ during LL phases after HL treatments: the relaxation was faster in OE‐NTR‐TRXf rosettes than in OE‐SGPS and *Col‐0* (Figures 5 and 6). Thus, the elevated activity of both NTRC and TRXf stimulates NPQ relaxation.

We have previously reported on dark reduction of the plastoquinone pool (chlororespiration) in OE‐NTRC leaves that is likely due to the increased activity of chloroplast NADH dehydrogenase‐like complex (NDH) (Nikkanen *et al*., 2018). Enhanced chlororespiration activates thylakoid STN7 protein kinase, uniquely resulting in phosphorylation of LHCII proteins in dark‐adapted OE‐NTRC leaves (Figure S4b) (Rintamäki *et al*., 2000; Nikkanen *et al*., 2018). Thus, we investigated, whether increased amount of TRXf in chloroplast can stimulate chlororespiration. Interestingly, an analysis of the phosphorylation status of PSII and LHCII proteins in fully developed leaves showed that LHCII proteins were phosphorylated only in dark‐adapted OE‐NTRC leaves, but not in dark‐adapted leaves of OE‐SGPS or OE‐NTR‐TRXf (Figure S4b), suggesting that only the TRX domain of NTRC is able to stimulate chlororespiration in dark‐adapted chloroplasts.

To analyse the effect of modified TRX activity on photosynthetic electron transfer further, we estimated the generation of proton motive force (*pmf*), proton flux over thylakoid membranes (*v*_H+_) and thylakoid conductivity to protons (*g*_H+_) in leaves of 25‐day‐old rosettes illuminated under fluctuating light (Figure S5). OE‐NTRC induced the higher generation of *pmf* and *v*_H+_, in both the LL and the HL phases in comparison with *Col‐0*, suggesting that OE‐NTRC is able to maintain higher electron transfer activity in thylakoids of OE‐NTRC chloroplasts than *Col‐0* throughout the treatment (Figures 5 and 6). The *pmf* and *v*_H+_ were lower in *ntrc* under the HL phase than in any other lines, and the *pmf* drifted up during illumination at LL (Figure S5a,c), indicating complex defects in the short‐term control of electron transfer activity in the absence of NTRC (see Nikkanen *et al*., 2018). In OE‐SGPS and OE‐NTR‐TRXf, generation of *pmf* and *v*_H+_ recovered to *Col‐0*‐like level, except that the generation of *pmf* in OE‐NTR‐TRXf was significantly lower after dark‐to‐LL transition. The first HL treatment recovered the generation of *pmf* in OE‐NTR‐TRXf to *Col‐0*‐level. Only in *ntrc* the *g*_H+_ was lower than in any other line throughout the fluctuating light treatment (Figure S5b), indicating that *ntrc* was not able to dissipate *pmf* and relax high NPQ as efficiently as the other lines during LL phases (Figures 5 and 6).

During the first 20 sec of LL illumination, NPQ was induced more rapidly in dark‐adapted OE‐NTRC than in any other line, but after this first peak NPQ gradually relaxed (Figures 6 and Figure S4a). Generation of high *pmf* preceded the rapid induction of NPQ in OE‐NTRC during the first seconds of illumination (Figure S5a), suggesting that the rapid induction of NPQ is due to increased electron transfer activity (Figure 6), which, in turn, induced increased acidification of the lumen at dark‐to light transitions and rapid induction of NPQ.

### Effect of leaf age on photosynthesis in transgenic lines

**Figure 7:**
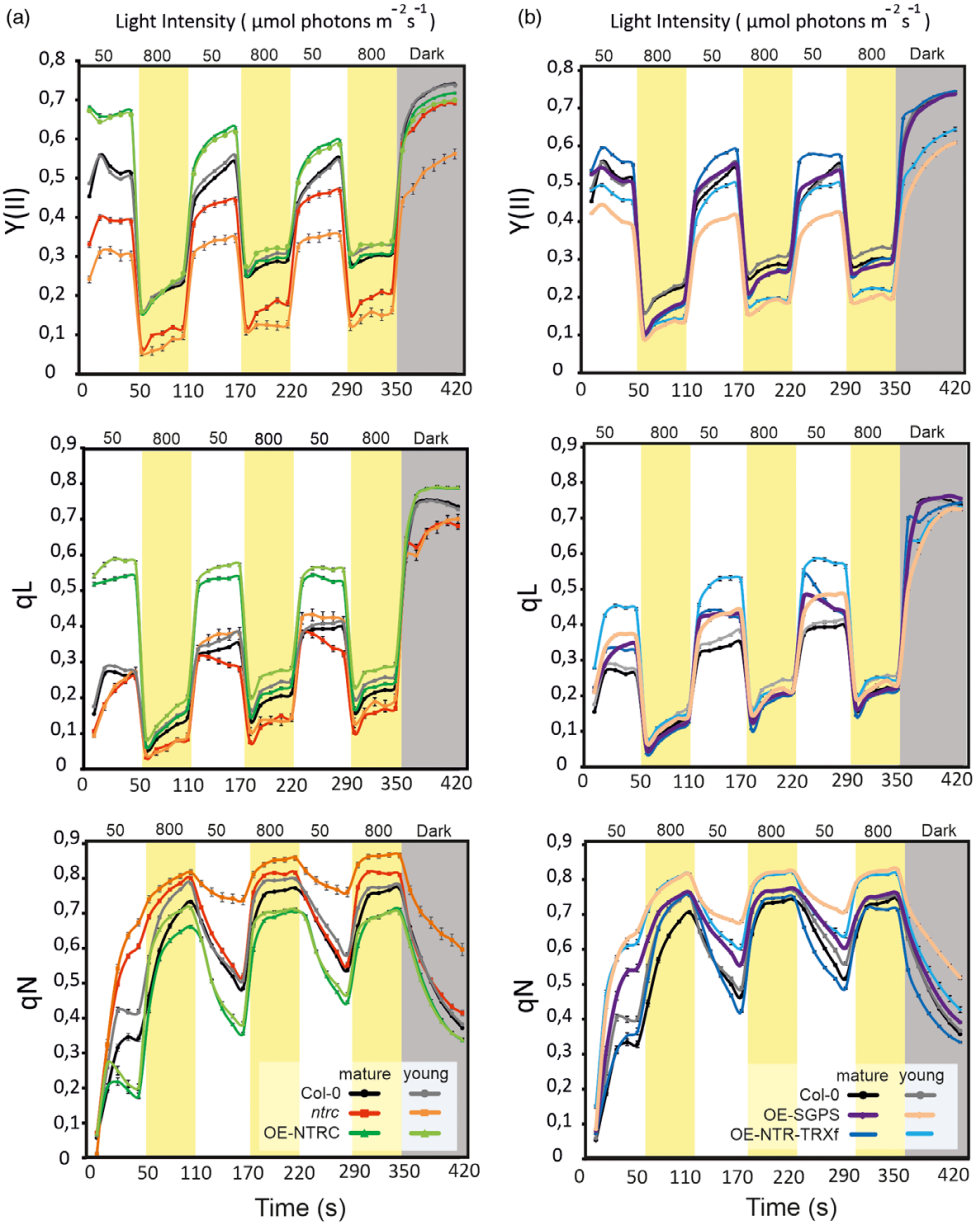
Photosynthetic parameters in young and mature leaves of transgenic lines exposed to fluctuating light. (a, b) Young and mature leaves of 40‐day old plants were exposed to low light/high light cycles as described in Figure 5. Chlorophyll *a* fluorescence was monitored during the treatments of plants and used for calculation of Photosystem II quantum yield (YII), photochemical quenching (qL) and non‐photochemical quenching (qN). .Comparison of photosynthetic parameters between young (grey, orange and light green line) and mature (black, red and green lines) leaves of *Col‐0, ntrc* and a transgenic line overexpressing NTRC (OE‐NTRC) during the exposure to fluctuating light (a) Comparison of photosynthetic parameters between young (grey, yellow and light blue lines) and mature (black, purple and cyan lines) leaves of *Col‐0,* OE‐NTR‐TRXf and OE‐SGPS (b). Mean values of 15–20 plants ± SEM are presented in the figure. SEM bars are only seen if the bar is higher than the symbol.

Photosynthetic parameters presented in Figures 3, 5 and 6 were measured from whole rosettes containing both young, developing leaves and mature leaves. The parameters determined for whole *Col‐0* rosettes under fluctuating light were relatively stable in plants of different ages (Figure 5), whereas variations in the values were observed with transgenic lines, particularly in *ntrc* and OE‐SGPS (Figure 5). This age‐dependent variation in OE‐NTRC, OE‐SGPS and OE‐NTR‐TRXf may be due to variable proportions of young and older leaves in the rosettes with different content of overexpressed protein (Figure 1). However, NTRC content cannot explain the variation observed in *ntrc* rosettes. To analyse the effect of leaf age on photosynthetic performance, we compared the photosynthetic parameters in young developing leaves with mature leaves within 40‐day‐old rosettes (Figures 7 and Figure S6).

No substantial differences were observed in the response of photosynthetic parameters to fluctuating light treatment between young and mature leaves of *Col‐0* and OE‐NTRC (Figure 7a). Induction and relaxation of NPQ at LL was also similar in young and old leaves of *Col‐0* or OE‐NTRC. In contrast, Y(II) was significantly lower in young than in mature leaves of *ntrc*, OE‐SGPS and OE‐NTR‐TRXf during the LL phases (Figure 7). Partial and full restoration of PSII quantum yield was recorded in mature leaves of *ntrc* and OE‐SGPS, respectively, during the LL phases of fluctuating light. In mature OE‐NTR‐TRXf leaves, Y(II) was even higher than in *Col‐0*. Accordingly, NPQ in the young leaves of OE‐SGPS and OE‐NTR‐TRXf responded to fluctuating light similarly to young leaves of *ntrc*: NPQ was strong at both LL and HL phases with lowest values recorded for young OE‐NTR‐TRXf leaves. However, *Col‐0*‐like relaxation of NPQ at the LL phases was observed in older *ntrc* and OE‐SGPS leaves, but only after the first HL treatment. The mature leaves of OE‐NTR‐TRXf responded to fluctuating light such as *Col‐0*, but the relaxation of NPQ was slightly faster during LL phases than in *Col‐0* (Figure 7b).

The changes in the photosynthetic responses in OE‐SGPS and OE‐NTR‐TRXf under fluctuating light may be explained by the higher accumulation of overexpressed proteins in mature than young leaves (Figure 1), which would increase the total activity of TRXf in leaves. How then to explain the recovery of photosynthetic parameters in mature leaves of *ntrc*? Likely, an adjustment of chloroplast redox components and/or redox state during maturation of *ntrc* leaves explains the restoration of photosynthesis activity. To investigate this possibility we analysed the amount and redox state of redox‐regulated chloroplast enzymes [TRXf, 2‐Cys PRXs, ascorbate peroxidases (APXs), redox‐regulated photosynthetic enzymes] in young and mature leaves of *Col‐0* and *ntrc* illuminated at GL (Figure S6). Both NTRC and TRXf content were slightly higher in mature than in young *Col‐0* leaves, as was the content of TRXf in older *ntrc* leaves. However, no remarkable differences between young and mature leaves could be observed in the redox state of TRXf in either *Col‐0* or *ntrc*. In addition, differences in other redox compounds in young and mature leaves of *ntrc* do not explain the partial recovery of photosynthesis in *ntrc*, as the content of antioxidants (2‐Cys PRXs, APX) and the redox state of 2‐Cys PRXs did not differ significantly between young and mature *ntrc* leaves. Of the tested proteins, only the content of cytosolic APX was substantially higher in young than in old leaves, both in *Col‐0* and *ntrc* (Figure S6d).

## DISCUSSION

### Molecular background of efficient photosynthesis in OE‐NTRC leaves

OE‐NTRC improves the quantum yield of photosynthesis, diminishes the induction of NPQ and accelerates its relaxation, particularly at irradiances limiting photosynthesis both in young and fully developed Arabidopsis rosettes (Figures 3, 4, 5, 6) (Nikkanen *et al*., 2018). It also induces constant activation of tested redox‐regulated chloroplast enzymes under steady illumination (Figure 4) (Nikkanen *et al*., 2016). The more efficient utilization of light energy and bypass of the reversible regulation of photosynthetic enzymes result in stimulation carbon fixation and of rosette growth (Figure 2) (Toivola *et al*., 2013; Nikkanen *et al*., 2016). Consequently, photosynthesis in OE‐NTRC plants is more tolerant of shading of leaves and light fluctuation.

Stimulation of photosynthesis in OE‐NTRC likely derives from an ability to oxidize PSI efficiently. OE‐NTRC has been shown to stimulate the activity of the following PSI electron acceptors: NTRC, the NDH complex in cyclic electron flow, the redox‐regulated enzymes in the CBB cycle, and 2‐Cys PRXs (Figures 3 and 5, 6, 7) (Nikkanen *et al*., 2016, 2018, 2019; Ojeda *et al*., 2018). The OE‐NTRC lines used in the experiments have 10–20 times higher NTRC content (Figure 1) (Toivola *et al*., 2013; Nikkanen *et al*., 2016) and fully reduced NTRC accumulates in leaves independently of light conditions (Nikkanen *et al*., 2018), indicating that more electrons are recruited to the NTRC system in comparison with *Col‐0*. More active NDH complex and constantly active redox‐regulated enzymes of CBB cycle help to balance the distribution of redox equivalents between sink and source at dark‐to‐light transitions and under rapid changes of light intensity. Yoshida and Hisabori (2016) reported that NTRC is a weak reductant of CBB cycle enzymes *in vitro* in comparison with the FTR system at wild‐type concentrations of enzymes and reductants. However, under light intensities limiting photosynthesis, when the FTR system is poorly activated (Yoshida *et al*., 2015; Nikkanen *et al*., 2016), impairment of photosynthesis is strongest in the absence of NTRC (Figures 3 and 5), suggesting that under these conditions NTRC is an indispensable reductant of photosynthetic enzymes even in *Col‐0*. In OE‐NTRC, the increased amount of NTRC protein likely compensates for the lower affinity of NTRC to the CBB enzymes and results in the higher accumulation of reduced enzymes in illuminated leaves (Figure 4) (Nikkanen *et al*., 2016).

NTRC, TRXy and TRXx have all been reported to re‐reduce 2‐Cys PRXs after oxidation by H_2_O_2_ (Collin *et al*., 2003; Pérez‐Ruiz *et al*., 2006; Bernal‐Bayard *et al*., 2014; Yoshida and Hisabori, 2016). However, NTRC is the primary reductant for 2‐Cys PRXs, as knockout of the *NTRC* gene increased the accumulation of H_2_O_2_ and oxidized forms of 2‐Cys PRXs in *ntrc* leaves (Pérez‐Ruiz *et al*., 2006; Kirchsteiger *et al*., 2009; Pulido *et al*., 2010; Nikkanen *et al*., 2016), indicating that TRXy and TRXx cannot compensate for lack of NTRC. Accordingly, 2‐Cys PRXs are strong acceptors of electrons in OE‐NTRC, because only reduced and half‐reduced 2‐Cys PRXs are observed in OE‐NTRC leaves (Figure 4) (Nikkanen *et al*., 2016). The high accumulation of reduced 2‐Cys PRXs may also derive from a lower reduction of O_2_ by PSI (resulting in lower production of superoxide/H_2_O_2_) because of the enhanced activity of other PSI acceptors in illuminated OE‐NTRC leaves.

Low lumen pH activates the energy‐dependent quenching component qE via protonation of the PsbS protein and activation the zeaxanthin synthesis in thylakoid membranes (Li *et al*., 2000; Jahns and Holzwarth, 2012; Sylak‐Glassman *et al*., 2014). NPQ induction was lower and relaxation faster in OE‐NTRC leaves in comparison with *Col‐0* despite higher generation of *pmf* and acidification of the lumen (Figures 3, 5 and Figure S5) (Nikkanen *et al*., 2019), suggesting that some other component(s) of NPQ than qE is affected by OE‐NTRC. Recently another, PsbS‐ and ΔpH‐independent quenching type called qH was introduced, which requires luminal lipocalin proteins and is repressed by a TRX‐like thylakoid protein SOQ1 that takes electrons from stromal TRX (Brooks *et al*., 2013; Malnoë *et al*., 2017). Enhanced amount of NTRC may persistently activate SOQ1, leading to repression of qH‐type NPQ and, when coupled to enhanced electron sink capacity on the acceptor side of PSI, improved utilization of light energy in photosynthesis, particularly under low and fluctuating light.

### Specificity and redundancy between NTRC, TRXf and TRXm in the regulation of photosynthesis

Proteomic and genetic approaches have revealed that chloroplast TRXs, particularly NTRC, TRXf and TRXm have several overlapping targets in photosynthetic reactions (Balmer *et al*., 2003; Lindahl and Kieselbach, 2009; Okegawa and Motohashi, 2015; Thormählen *et al*., 2015; Nikkanen *et al*., 2018). OE‐NTRC in Arabidopsis enhanced biomass production (Toivola *et al*., 2013) and OE‐TRXf in tobacco increased specific leaf weight and starch content (Sanz‐Barrio *et al*., 2013), whereas OE‐TRXm impaired growth and decreased chlorophyll content in tobacco leaves (Rey *et al*., 2013; Sanz‐Barrio *et al*., 2013; Ancin *et al*., 2018). Thus, overexpressed NTRC and TRXf have a parallel and positive role, and overexpressed TRXm an antagonist role in the regulation of photosynthesis and its productivity. Accordingly, antagonistic functions of the TRXm isoforms relative to NTRC/TRXf in photosynthesis have been reported. TRXm1 and TRXm2 have been observed to affect PSII and PSI quantum yields in LL negatively (Thormählen *et al*., 2017), and TRXm4 has been proposed to inhibit NDH‐dependent CEF (Courteille *et al*., 2013), while these processes are positively affected by NTRC.

In this study, we investigated the redundancy between NTRC and TRXf in the regulation of photosynthesis by overexpressing NTRC and TRXf on *ntrc* background. The rise of TRXf content in transgenic lines was never able to complement retarded growth in the absence of NTRC fully, indicating that NTRC has non‐redundant functions that are not replaceable by TRXf (Figure 2). This is compatible with the observation that while growth and photosynthesis are only slightly affected in the *trxf1 trx2* double mutant but significantly deteriorated in *ntrc*, they are impaired even further in *trxf1 ntrc* double mutant (Thormählen *et al*., 2015). The data presented here support previous reports showing that the redox targets primarily regulated by NTRC include 2‐Cys PRXs (Pérez‐Ruiz *et al*., 2006; Pulido *et al*., 2010) as well as the NDH complex, as only *Col‐0* and OE‐NTRC lines are able to keep a substantial amount of 2‐Cys PRXs in the reduced form in illuminated leaves (Figure 4), and enhancement of NDH‐dependent chlororespiration (Nikkanen *et al*., 2018) was only observed in OE‐NTRC leaves (Figure S4a).

The lack or overexpression of NTRC has the highest impact on photosynthesis under conditions where the FTR‐dependent system is less active (Yoshida *et al*., 2015; Nikkanen *et al*., 2016; Thormählen *et al*., 2017), i.e., during dark‐to‐light transitions, under irradiances lower than GL and under fluctuating light. Under these conditions, the effect of extra content of TRXf showed a clear dose effect: the higher the potential amount of TRXf protein was in chloroplasts, the better recovery of photosynthetic function was recorded (Figures 3, 5 and 6). This indicates that TRXf is not a primary regulator of photosynthesis upon light changes but can compensate for the absence of NTRC if present at higher concentrations, as in mature leaves (Figure 7). The dose effect of TRXf on NPQ was most clearly seen in the relaxation of NPQ after HL phases of fluctuating light (Figures 6 and 7). The higher the content the TRXf was, the faster the relaxation rate of NPQ at LL was detected. The recovery of NPQ relaxation in OE‐SGPS and OE‐NTR‐TRXf was partly enabled by the recovery of the proton conductivity of the thylakoid membrane (*g_H+_*) to *Col‐0* level (Figure S5). Interestingly, however, reduction of the CF1γ subunit of the ATP synthase in LL was not recovered in OE‐SGPS or in OE‐NTR‐TRXf (Figure 4). This suggests that the increase in TRXf activity affected a redox‐sensitive component(s) controlling the activity of the ATP synthase other than the CF1γ subunit. Alternatively, the ion channels and transporters on the thylakoid membrane such as the K^+^/H^+^ exchanger KEA3, or the Cl^−^ channels CLCe and VCCN1 may be targeted to redox regulation. Mutants lacking these transporters have defects in regulation of the *pmf* and/or induction and relaxation of NPQ (Armbruster *et al*., 2016; Herdean *et al*., 2016a; Herdean *et al*., 2016b; Spetea *et al*., 2017).

In transgenic lines lacking NTRC, HL treatment of the leaves restores the level of reduced PRK independently of the amount of TRXf (Figure 4). The HL activation of PRK may be due to activation of other FTR‐dependent TRXs, such as TRXm isoforms, from which TRXm2 has reported to become substantially reduced only under irradiances higher than GL (Yoshida *et al*., 2015). Accordingly, the substantial decrease in content of reduced FBPase and SBPase was detected in *trxf1 trxf2* double mutant illuminated at LL and GL, whereas higher light treatments restored the reduction of these enzymes (Yoshida *et al*., 2015; Naranjo *et al*., 2016a). Furthermore, OE‐NTRC slightly increased the content of reduced FBPase in illuminated leaves (Nikkanen *et al*., 2016). FBPase and SBPase are poor substrates for NTRC and TRXm2 *in vitro* (Yoshida *et al*., 2015; Yoshida and Hisabori, 2016), suggesting that these enzymes are primarily regulated by TRXf, but can be reduced by TRXm2 or NTRC under conditions where the activity of these TRXs relative to TRXf has risen. These observations suggest that redox‐regulated enzymes of the CBB cycle can be targeted to different TRXs and TRX systems depending on light intensity, which is likely beneficial under fluctuating light.

In conclusion, we propose that the NTRC system is involved in the control of electron transfer activity in the thylakoid membrane via primarily regulating NDH‐dependent CEF, and conditionally regulating NPQ, and the activity of ATP synthase and PRK at LL and at light transitions. In the latter case, endogenous activity of the FTR‐dependent TRXs in *Col‐0* cannot compensate for NTRC, while increased TRXf content coupled to NADPH‐dependent reductase activity partially or fully recovers the control of the ATP synthase and NPQ in the mutants lacking NTRC. At dark‐to‐light transitions, redox regulation of NPQ, NDH‐dependent CEF and ATP synthase in the thylakoid membrane balances electron donor and acceptor sites of PSI during the first minute of illumination. It also allows the ATP production that together with the redox activation of PRK initiates the CBB cycle via synthesis of ribulose‐1,5‐bisphosphate, a substrate of Rubisco. TRX and ATP are also involved in the activation of Rubisco via Rubisco activase (Zhang and Portis, 1999; Naranjo *et al*., 2016a), suggesting that these first CBB reactions can be quickly switched on by illumination before activation of other redox‐regulated enzymes in later steps of the CBB cycle, such as FBPase and SBPase.

### Antioxidant capacity and redox regulation of photosynthesis

It was recently suggested that the capacity to activate redox‐regulated photosynthetic enzymes depends on the amount of redox equivalents used for removing H_2_O_2_ by 2‐Cys PRXs in chloroplasts (Pérez‐Ruiz *et al*., 2017; Vaseghi *et al*., 2018). According to this hypothesis, in the absence of NTRC a large proportion of redox equivalents is removed from the activation of photosynthetic enzymes and used for reduction of 2‐Cys PRXs, which impairs photosynthesis and reduces the growth of *ntrc* (Pérez‐Ruiz *et al*., 2017). Recovery of growth in *ntrc‐∆2cp* triple mutants lacking NTRC and having a decreased amount of 2‐Cys PRXs supports the hypothesis (Pérez‐Ruiz *et al*., 2017). The growth and photosynthetic phenotype of OE‐NTRC line may also support this hypothesis, as rosette growth of OE‐NTRC is stimulated as more electron equivalents are recruited to both TRX systems to keep the redox‐regulated enzymes in reduced form in illuminated leaves (NTRC, TRXf, PRK, 2‐Cys PRXs) (Figure 4; Nikkanen *et al*., 2016, 2018). However, overexpression of the NTRC_SGPS_ form on the *ntrc* background recovered rosette growth in comparison with *ntrc*, and even more recovery was observed by overexpression of the chimeric NTR‐TRXf enzyme (Figure 2), although no recovery was observed in the steady‐state proportion of reduced 2‐Cys PRXs, CF1γ or PRK in OE‐NTR‐TRXf or OE‐SGPS leaves illuminated at GL (Figure 4). On the contrary, in OE‐SGPS, the redox balance of 2‐Cys PRXs was even more oxidized at GL than in *ntrc* (Figure 4, Nikkanen *et al*., 2016). These results suggest that the link between growth capacity and chloroplast TRX systems is complex and cannot directly be determined by the size and/or the redox status of the 2‐Cys PRXs pool.

## EXPERIMENTAL PROCEDURES

### Plant material and growth conditions

Wild‐type A. *thaliana* of the ecotype (*Col‐0*), T‐DNA insertion mutant line of *NTR*C (At2g41680) (*ntrc*, SALK_096776) (Alonso *et al*., 2003; Lepistö *et al*., 2009), as well as homozygous plants of the OE‐NTRC and OE‐SGPS lines (Toivola *et al*., 2013; Nikkanen *et al*., 2016) were used in the experiments. To generate the OE‐NTRC‐TRXf line, the NTR domain of NTRC (amino acids 1–429) with a flexible linker region at the C‐terminus was amplified as an *Nco*I–*Xma*I fragment from the *NTRC* gene in the OE‐NTRC.pGWR8 construct (Toivola *et al*., 2013) with forward (*CTGCCATGGATATGGCTGCGTCTCCCAAGA*) and reverse (*CGCCCGGGCTTGTGCTTTGTAAGAGTGATG*) primers. The NTR domain was then fused with the coding sequence of the Arabidopsis *TRXf1* gene (At3g02730) containing *Xma*I–*Bam*HI restriction sites. TRXf1 was amplified with a forward (*CGCCCGGGTGTAGCTTAGAAACCGTTAATG*) and reverse (CGGGATCCTCATCCGGAAGCAGCAGAC) primers to omit the *TRXf1* chloroplast signal sequence (predicted transit peptide of 57 amino acids). The NTR‐TRXf1 fragment was then inserted as an *Nco*I*–Bam*HI fragment into the pGWR8 expression vector (Rozhon *et al*., 2010), where the chimeric protein is expressed under the cauliflower mosaic virus (CaMV) 35S promoter. Sequenced construct was introduced to A*grobacterium tumefaciens* strain GV3101 by electroporation, and transformed into the *ntrc*‐knockout line of Arabidopsis by floral dipping (Clough and Bent, 1998). T0 seeds were screened with 50 μg ml^−1^ of kanamycin, and five lines showing recovery of the *ntrc* mutant phenotype were selected for further screening. Overexpression of chimeric *NTR‐TRXf* gene was confirmed by immunoblotting with NTRB‐ and TRXf‐specific antibodies.

For immunoblot analyses as well as measurement of ECS plants were grown in 1:1 mixture of soil and vermiculite under 200 μmol photons m^−2^ sec^−1^ at 23°C in a short day (8 h light/16 h dark) photoperiod using Philips TL‐D 36W/840a (Eindhoven, the Netherlands) fluorescent tubes as light sources.

For the phenotypic analysis, seeds were grown on soil with 50% peat and 50% vermiculite. Seeds were stratified at +4°C for three nights and transferred to a FytoScope growth chamber (Photon System Instruments, PSI, Drasov, Czech Republic). Plants were grown at 200 μmol photons m^−2^ sec^−1^ (GL) (LI‐COR Quantum Sensor LI‐190R; LI‐COR Biosciences ®, Lincoln, NE, USA) with 8 h light/16 h darkness at 22°C and a relative air humidity of 60%. The humidity of the soil was set at 70% and maintained by daily weighing and watering. To determine the photosynthetic parameters in seedlings, plants were grown on 0.8% agar plates with 0.5× Murashige and Skoog basal salt mixture.

### High‐throughput plant phenotyping platform

The phenotypic characterization of the Arabidopsis lines at NaPPI was done as described in Pavicic *et al*. (2017). The NaPPI small plant unit (PlantScreenTM Compact System) is in a controlled environment FytoScope Walk‐In chamber (PSI). Within the system, trays with 20 plants each were transported on conveyor belts between the light‐isolated visible RGB and chlorophyll fluorescence imaging cabinets, weighing and watering station and the dark/light acclimation chamber with regulated light intensities from 0 to 700 μmol photons m^−2^ sec^−1^ (Figure S1). RGB images (resolution 2560 × 1920 pixels) of individual trays with 20 plants were captured using the GigE uEye UI‐5580SEC/M 5 Mpx Camera (IDS, Obersulm, Germany) with the SV‐0814H lens, with LED light conditions, plant position and camera settings fixed throughout the experiments. The images were pre‐processed online as described in Awlia *et al*. (2016) to allow collecting binary and RGB data for each plant.

The Chl*a* fluorescence measurements were acquired using an enhanced version of the FluorCam FC‐800MF pulse amplitude modulated chlorophyll fluorometer (PSI, Czech Republic). The Chl*a* fluorescence imaging unit has been described in Awlia *et al*. (2016), and features a 1/2" monochromatic sensor of 720 × 560 pixels resolution and a lens type Lensagon CY0314. The FC illumination panel (FluorCam SN‐FC800‐195) has a pulse‐modulated short duration red–orange flashes (620 nm), a red–orange actinic light (620 nm) with maximum light intensity of 300 μmol photons m^−2^ sec^−1^, a cool white actinic light with maximum light intensity of 500 μmol photons m^−2^ sec^−1^ and a saturating light pulse with a maximum light intensity of 3000 μmol photons m^−2^ sec^−1^.

To analyse the photosynthetic performance of the lines, two different protocols were applied weekly from 10 to 50 days after stratification. In the light curve protocol (Figure S1b), plants were transferred after dark adaptation for 20 min to increasing light intensities (20, 50, 100, 200, 500, 700 and 800 μmol photons m^−2^ sec^−1^) and kept for 1 min under each condition. A saturating pulse of 2000 μmol photons m^−2^ sec^−1^ was applied at the end of illumination at each light intensity.

To study the response to fluctuating light, a quenching protocol (Figure S1c) was used. In this protocol, plants were dark adapted for 20 min and a saturating pulse of light was applied for the determination of *F*_v_/*F*_m_. Then, plants were illuminated with 50 μmol photons m^−2^ sec^−1^ (LL) with saturating pulses applied every 10 sec. After 1 min, the light intensity was increased to 800 μmol photons m^−2^ sec^−1^ (HL) for 1 min with a saturating pulse every 10 sec. The LL–HL cycle was repeated three times. After cessation of illumination, saturating pulses were applied to determine quenching relaxation.

The fluorescence data were obtained for a whole rosette, stored in a central database (http://doi.org/10.23728/b2share.a5f7ae90634044e3aebef2c9e10a4c4d; http://doi.org/10.23728/b2share.6b8de1d52c97486cbb5a1023ffcafc13) and analysed using the program FluorCam7 (PSI). The phenotypic characterization of the lines was repeated three times (20 plants each) with a representative data set presented in the figures. Student’s *t*‐test (*P* < 0.05) was used to confirm differences between lines. The parameters Y(II), qN and qL were calculated as described by Roháček (2002) and Kramer *et al*. (2004).

### Protein extraction, sodium dodecyl sulphate–polyacrylamide gel electrophoresis and Western blotting

Extraction of leaf proteins, determination of protein content, separation of proteins by sodium dodecyl sulphate–polyacrylamide gel electrophoresis and Western blotting were done as described previously (Toivola *et al*., 2013; Nikkanen *et al*., 2016). After blotting, membranes were probed overnight at 4°C with primary antibodies raised against PRK (AS07 257; Agrisera AB, Vännas, Sweden), FBPase (kindly provided by Dr M. Sahrawy, CSIC, Spain), CF1γ (AS08 312; Agrisera), 2‐Cys PRXs and NTRB (kindly provided by Prof. F. J. Cejudo, Institute of Plant Biochemistry, University of Sevilla), TRXf1/2 (AS14 2808; Agrisera), APX (AS08 368; Agrisera), ADP‐glucose pyrophosphorylase (AS11 1739; Agrisera) and NTRC (Lepistö *et al*., 2009). A horseradish peroxidase‐conjugated goat antirabbit secondary antibody (AS09 602; Agrisera) was applied for 2 h. All immunoblots shown are representative of at least three biological replicates of similar results.

### Alkylation of protein thiols

Arabidopsis rosettes were illuminated for 2 h or dark‐adapted for 30 min before freezing the leaves in liquid nitrogen and storage at −80°C until use. Trichloroacetic acid (TCA) precipitation and thiol alkylation with methoxypolyethylene glycol maleimide (MAL‐PEG) or 4‐acetamido‐4′‐maleimidylstilbene‐2,2′‐disulfonic acid were performed according to Nikkanen *et al*. (2016). Briefly, TCA precipitated and acetone‐washed proteins were treated with *N*‐ethylmaleimide (Sigma‐Aldrich, St. Louis, MI, USA) to block protein thiols that were present before isolation of the proteins. This *N*‐ethylmaleimide treatment induces only a slight increase in the molecular weight of a protein. Then, samples were incubated with 100 mm DTT to reduce residual disulphide bonds in proteins, after which TCA precipitation and acetone washing of the samples were repeated, and the samples were treated with 10 mm of MAL‐PEG (molecular weight 5000; Sigma‐Aldrich). MAL‐PEG binds to protein thiols produced by DTT treatment and increases the molecular mass of the protein by 5 kDa per MAL‐PEG bound. Following this procedure the proteins that were oxidized (contained disulphide) *in vivo*, were labelled with MAL‐PEG. Owing to the MAL‐PEG‐induced increase in molecular mass, oxidized forms of proteins can be separated from the reduced form in sodium dodecyl sulphate–polyacrylamide gel electrophoresis.

### Measurement of ECS

To measure the magnitude and kinetics of *pmf* formation and to determine the conductivity of the ATP synthase to protons, changes in the ECS (P515) signal were recorded with a Dual‐PAM‐100 spectrometer and its P515/535 accessory module (Walz, Effeltrich, Germany) (Schreiber and Klughammer, 2008; Klughammer *et al*., 2013). A measuring light at a 2000‐Hz pulse frequency was used in all ECS measurements as described in Nikkanen *et al*. (2018). Dark intervals of 250 msec were applied at time‐points specified in Figure S5 to determine the magnitude of light‐induced *pmf* (ECS_T_), and to calculate the conductivity parameters of the ATP synthase (*g*_H_+) as well as the proton flux parameter (*v*_H_+), as described previously (Cruz *et al*., 2001; Cruz *et al*., 2005; Nikkanen *et al*., 2018).

## AUTHOR CONTRIBUTIONS

MGD, LN, KH and ER designed the research; MGD, LN and JT performed the experiments; MGD and LN analysed the data; MGD, LN, KH and ER wrote the paper.

## CONFLICT OF INTERESTS

The authors declare that they have no competing interests.

## Supporting information


**Figure S1**. Experimental procedures of the phenotypic analysis.
**Figure S2**. Seedling phenotypes of the transgenic lines.
**Figure S3**. Proton conductivity of the ATP synthase at dark‐to‐light transition.
**Figure S4**. Induction of NPQ at dark‐to‐light transition and accumulation of phosphorylated thylakoid proteins in dark‐adapted and illuminated leaves.
**Figure S5**. Generation of the proton motive force (*pmf*) in fluctuating light.
**Figure S6**. Redox states of chloroplast proteins in young and mature leaves of *Col‐0* and *ntrc*.

## Data Availability

The original data files of Chl*a* fluorescence measurements are found at a central database (http://doi.org/10.23728/b2share.a5f7ae90634044e3aebef2c9e10a4c4d; http://doi.org/10.23728/b2share.6b8de1d52c97486cbb5a1023ffcafc13).
