# Supplementary material for: Two chloroplast thioredoxin systems differentially modulate photosynthesis in Arabidopsis depending on light intensity and leaf age
Source: Plant J. 2020 Aug 31;104(3):718–34. doi: 10.1111/tpj.14959 (PMC7693050; doi:10.1111/tpj.14959)
Supplement: Supplementary file 1 — Figure S1. Experimental procedures of the phenotypic analysis. Figure S2. Seedling phenotypes of the transgenic lines. Figure S3. Proton conductivity of the ATP synthase at dark‐to‐light transition. Figure S4. Induction of NPQ at dark‐to‐light transition and accumulation of phosphorylated thylakoid proteins in dark‐adapted and illuminated leaves. Figure S5. Generation of the proton motive force (pmf) in fluctuating light. Figure S6. Redox states of chloroplast proteins in young and mature leaves of Col‐0 and ntrc. [file TPJ-104-718-s001.docx]

**Two chloroplast thioredoxin systems differentially modulate photosynthesis in Arabidopsis depending on light intensity and leaf age**

Manuel Guinea Diaz, Lauri Nikkanen, Kristiina Himanen, Jouni Toivola, and Eevi Rintamäki

**SUPPORTING INFORMATION**

**Figure S1**

**
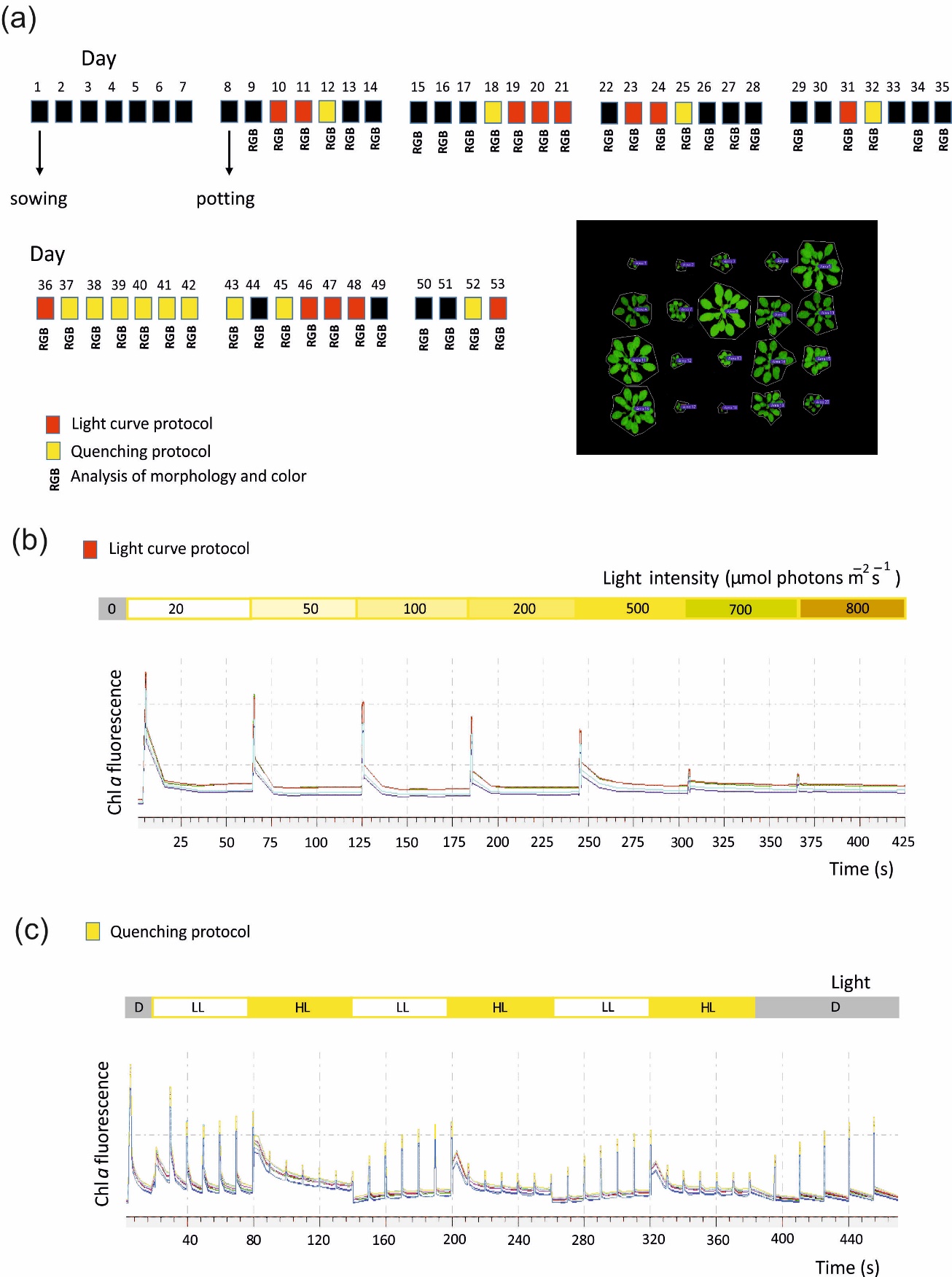
**

**Figure S1.** Experimental procedures of the phenotypic analysis.

(a) After stratification at +4 °C for three nights the trays were transferred to the “walk-in” growth chamber. Seven days after stratification (DAS) the seedlings were moved to their own pots and placed on the analysis trays. The dates of the analysis are marked in the figure: quenching analysis (yellow squares), light curve analysis (red squares), and photos of the plants used for colour analysis (RGB). (b) Schematic representation of the light curve protocol: after dark adaptation of 20 min, trays were illuminated with increasing light intensities (20, 50, 100, 200, 500, 700 and 800 μmol photons m^-2^ s^-1^) for 1 min, after which the saturating pulse was applied and Chl*a* fluorescence recorded. (c) Schematic representation of the quenching protocol. The trays were dark- adapted for 20 min and exposed to three consecutive cycles of low light (LL)/high light (HL) (LL: 50 μmol photons m^-2^ s^-1^; HL: 800 μmol photons m^-2^ s^-1^) before switching the light off. Saturating pulses were applied every 10 seconds and Chl*a* fluorescence was measured to determine photosynthetic parameters under the experimental conditions.

**Figure S2**


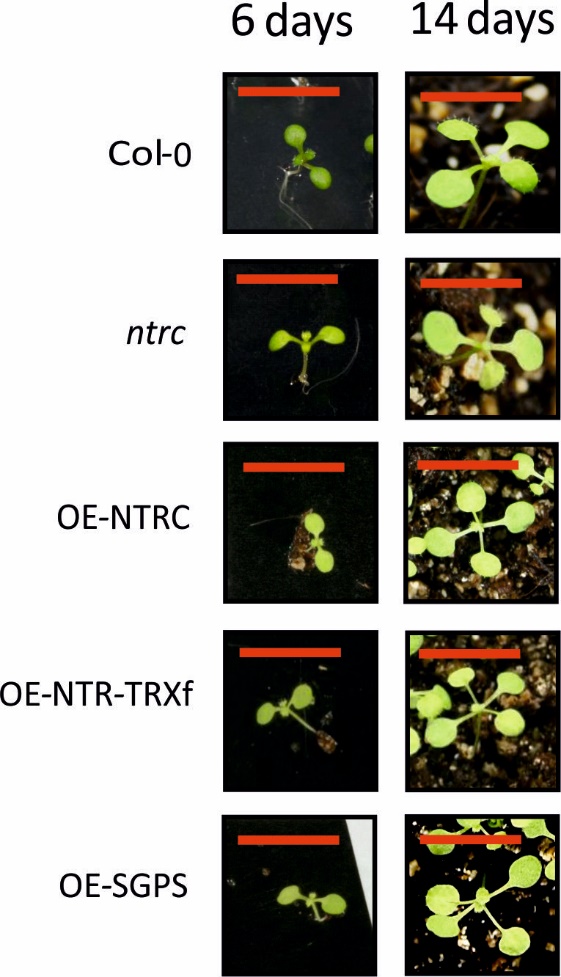


**Figure S2.** Seedling phenotypes of the transgenic lines.

Seedlings of Arabidopsis wild-type (*Col-0*), *NTRC* knockout mutant (*ntrc*) and transgenic lines overexpressing modified TRX systems (*OE-NTRC, OE-NTR-TRXf, OE-SGPS*) were grown in short day conditions (8h light/16h dark) at 200 μmol photons m^-2^ s^-1^. The photos were taken at ages of 6, and 14 days after stratification. Scale bar is 1.5 cm.

**
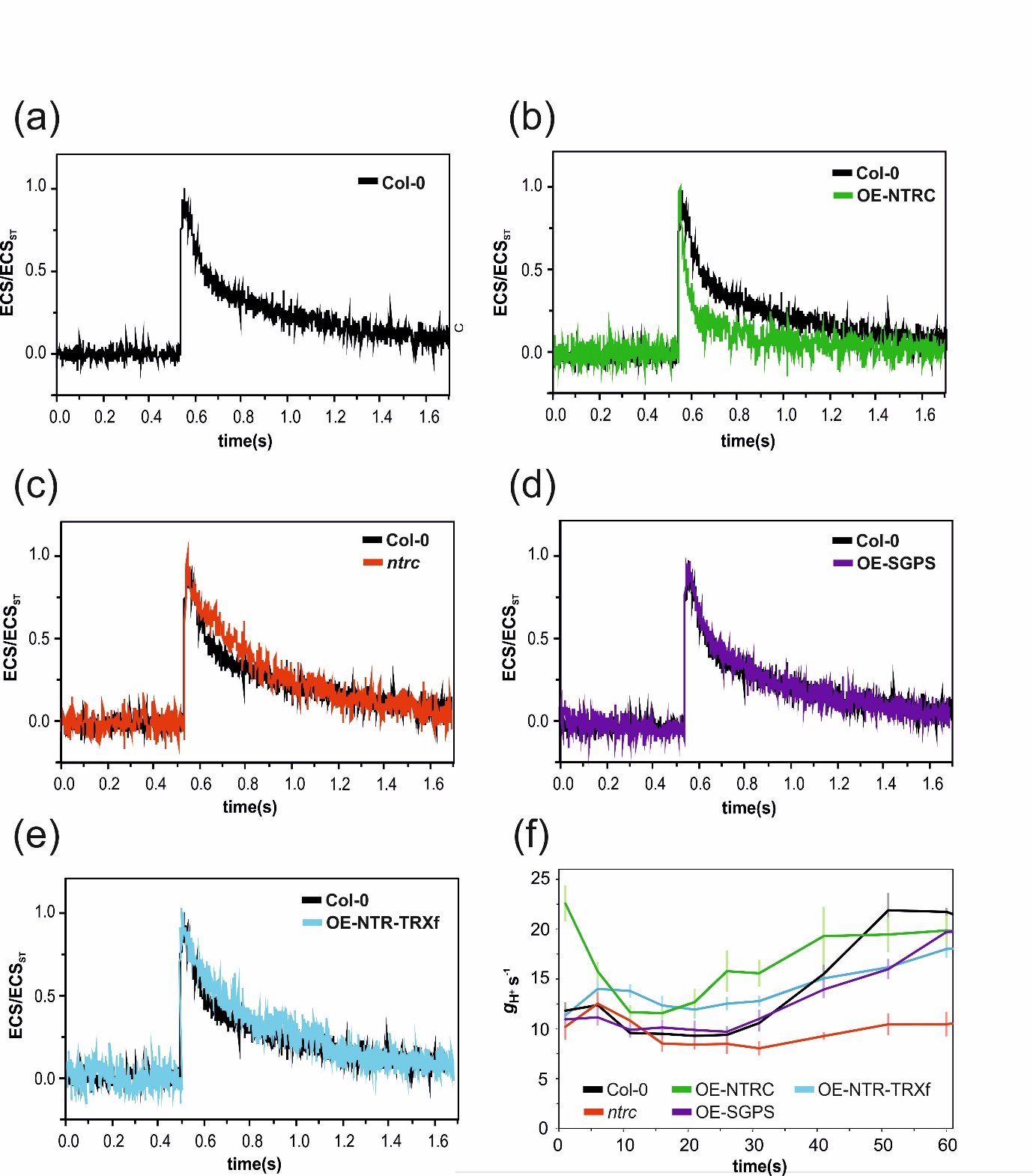
Figure S3**

**Figure S3.** Proton conductivity of the ATP synthase at dark to light transition.

(a-e) Estimation of ATP synthase conductivity in dark-adapted leaves of 25 days old transgenic lines in comparison to *Col-0*. *Col-0* (black), *ntrc* (red), OE-NTRC (green), OE-NTR-TRXf (cyan), and OE-SGPS (purple) leaves were dark-adapted for 30 min before measurement of the decay of the electrochromic shift signal (ECS) after a 20 µs single-turnover flash of 10,000 μmol photons m^-2^ s^-1^. The values were normalized with the maximal magnitude of the flash-induced ECS signal (ECS_ST_) (Kramer and Crofts, 1989). Representative traces from 5–8 measurements from individual leaves are shown. (f) Conductivity of the ATP synthase to protons (g_H+_) during the first minute of a transition from dark to low light (51 μmol photons m^-2^ s^-1^). *Col-0*, OE-NTRC, *ntrc*, OE-SGPS, and OE-NTR-TRXf leaves were dark-adapted for 30 min before switching the LL on and estimation of *g_H+_* as the inverse of the time constant of a first‐order fit to the decay of the ECS signal during 250 ms dark intervals. The values shown are averages from 5–8 measurements from individual leaves ± SEM.

**Figure S4**

**
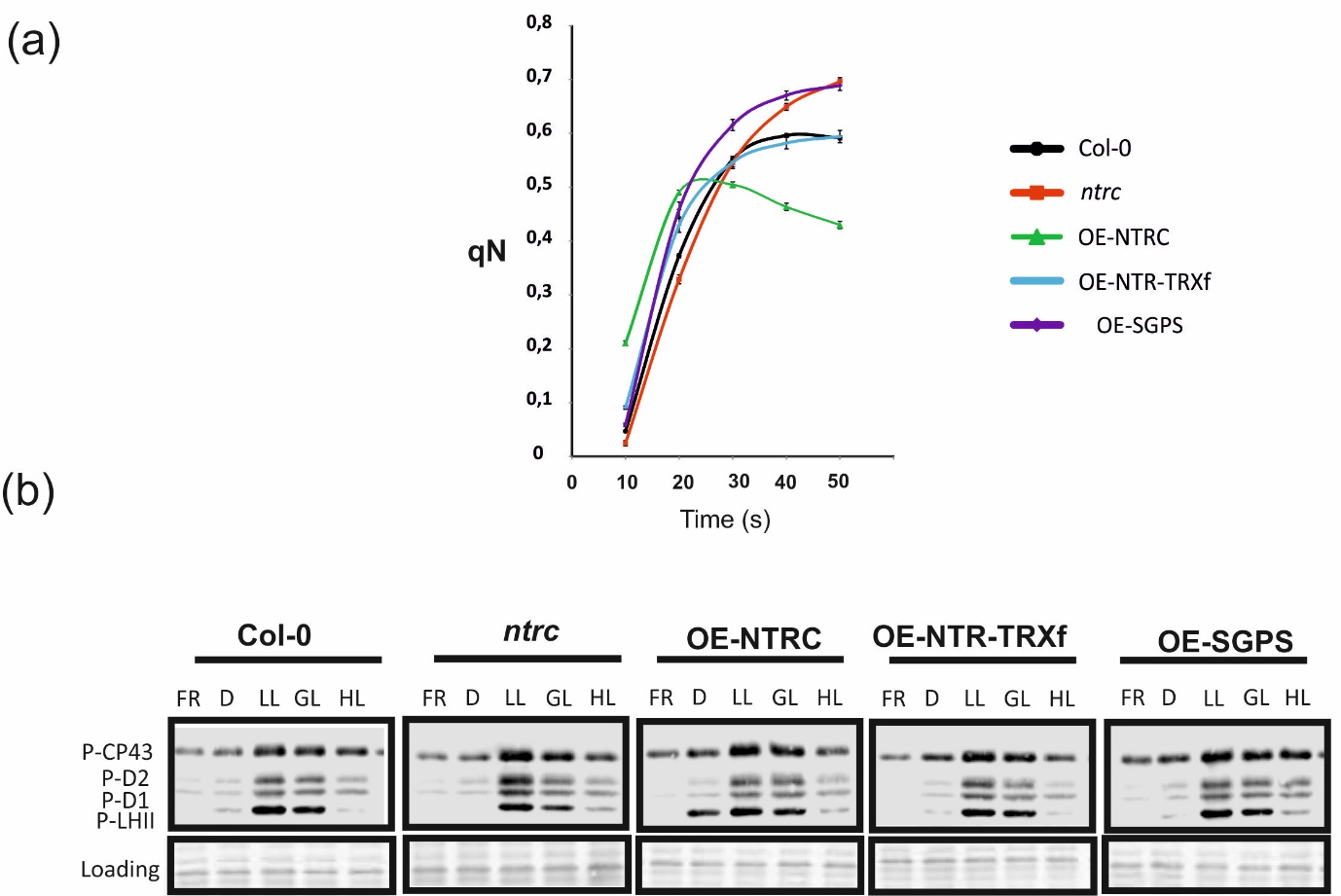
**

**Figure S4.** Induction of NPQ at dark to light transition and accumulation of phosphorylated thylakoid proteins in dark-adapted and illuminated leaves.

(a) NPQ (qN) induction in dark to low light (50 μmol photons m^-2^ s^-1^) transition in the 25 days old lines *Col-0* (black dots), *ntrc* (red squares), OE-NTRC (green triangles), OE-NTR-TRXf (cyan lines) and OE-SGPS (purple diamonds). (b) Thylakoid protein phosphorylation in dark-adapted (D) and illuminated transgenic lines. Plants were dark-adapted overnight (D) or illuminated under far red light of 730 nm for 2 h (FR) before illumination at 50 (LL), 200 (GL) or 800 (HL) μmol photons m^-2^ s^-1^ for 2 h. Thylakoid membrane proteins were separated by SDS PAGE and immunoblotted as described in Nikkanen *et al.* (2016). The membrane was probed with antibody raised against phosphothreonine (P-Thr) (New England Biolabs). P-Thr antibody recognizes phosphorylated form of 43 kDa chlorophyll a binding protein (CP43), reaction center proteins of Photosystem II (D1 and D2) and light harvesting proteins of Photosystem II (LHCII). Licor Revert staining was used as loading control.

**Figure S5**

**
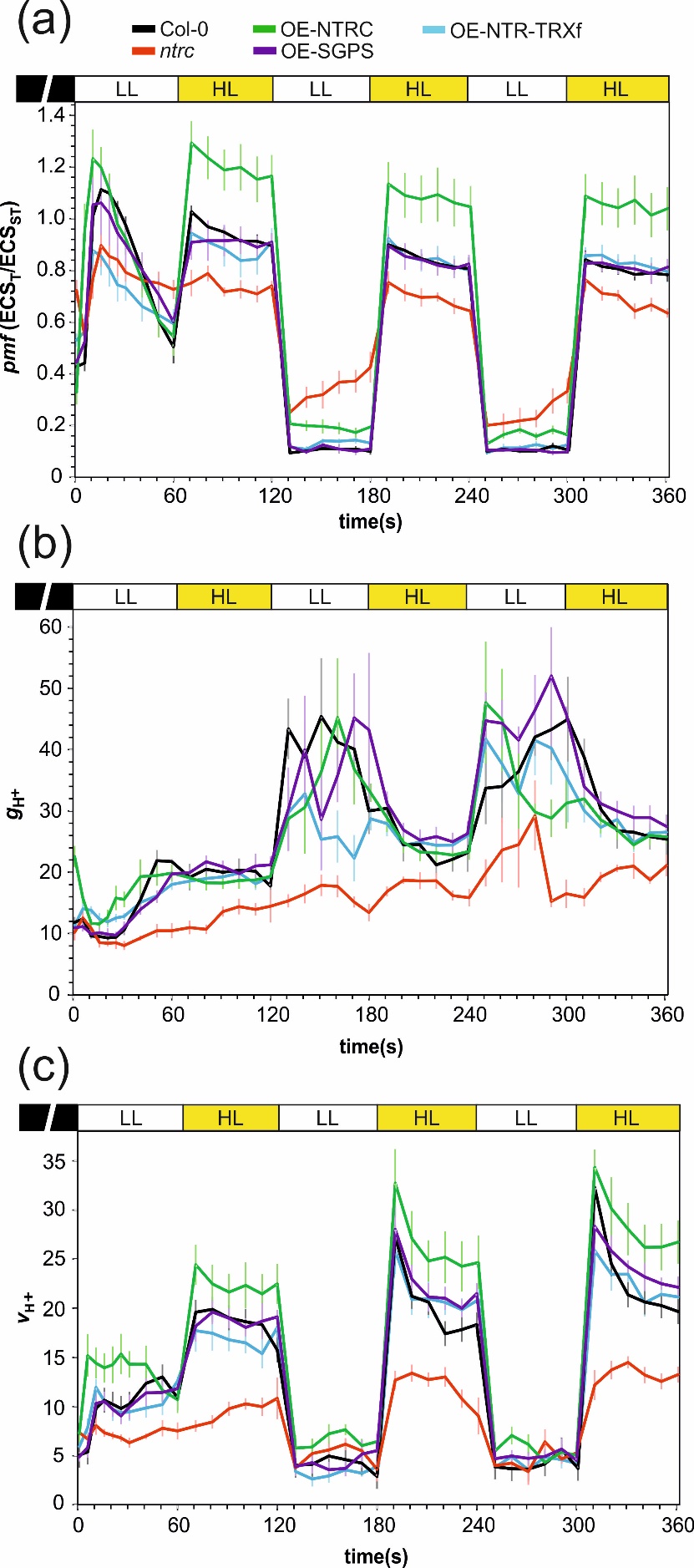
**

**Figure S5.** Generation of the proton motive force (*pmf*) in fluctuating light.

(a) Magnitude of the *pmf* during a light treatment where light intensity fluctuates between 1 min periods of low (LL, 51 μmol photons m^-2^ s^-1^) and high light (HL, 825 μmol photons m^-2^ s^-1^). Before the experiment, leaves from 25 days old *Col-0* (black), *ntrc* (red), OE-NTRC (green), OE-NTR-TRXf (cyan), and OE-SGPS (purple) plants were dark-adapted for 30 min. The *pmf* was determined from the light-induced change in the ECS signal (ECS_T_) and normalized with the magnitude of ECS induced by a 20 μs saturating single-turnover flash (ECS_ST_) given before turning on actinic light (Kramer and Crofts, 1989). The values shown are averages from 5–8 measurements from individual leaves ± SEM. (b) Conductivity of the ATP synthase to protons (*g*_H+_) during the fluctuating light regime in (a). The *g*_H+_ was determined as the inverse of the time constant of a first‐order fit to the decay of the ECS signal during 250 ms dark intervals. The values shown are averages from 5–8 measurements from individual leaves ± SEM. (c) Proton flux over the thylakoid membrane (*v*_H+_) during the fluctuating light experiment in (a) and (b). The *v*_H+_ was calculated as *pmf x g_H+_*. The values shown are averages from 5–8 measurements from individual leaves ± SEM.

**Figure S6**

**
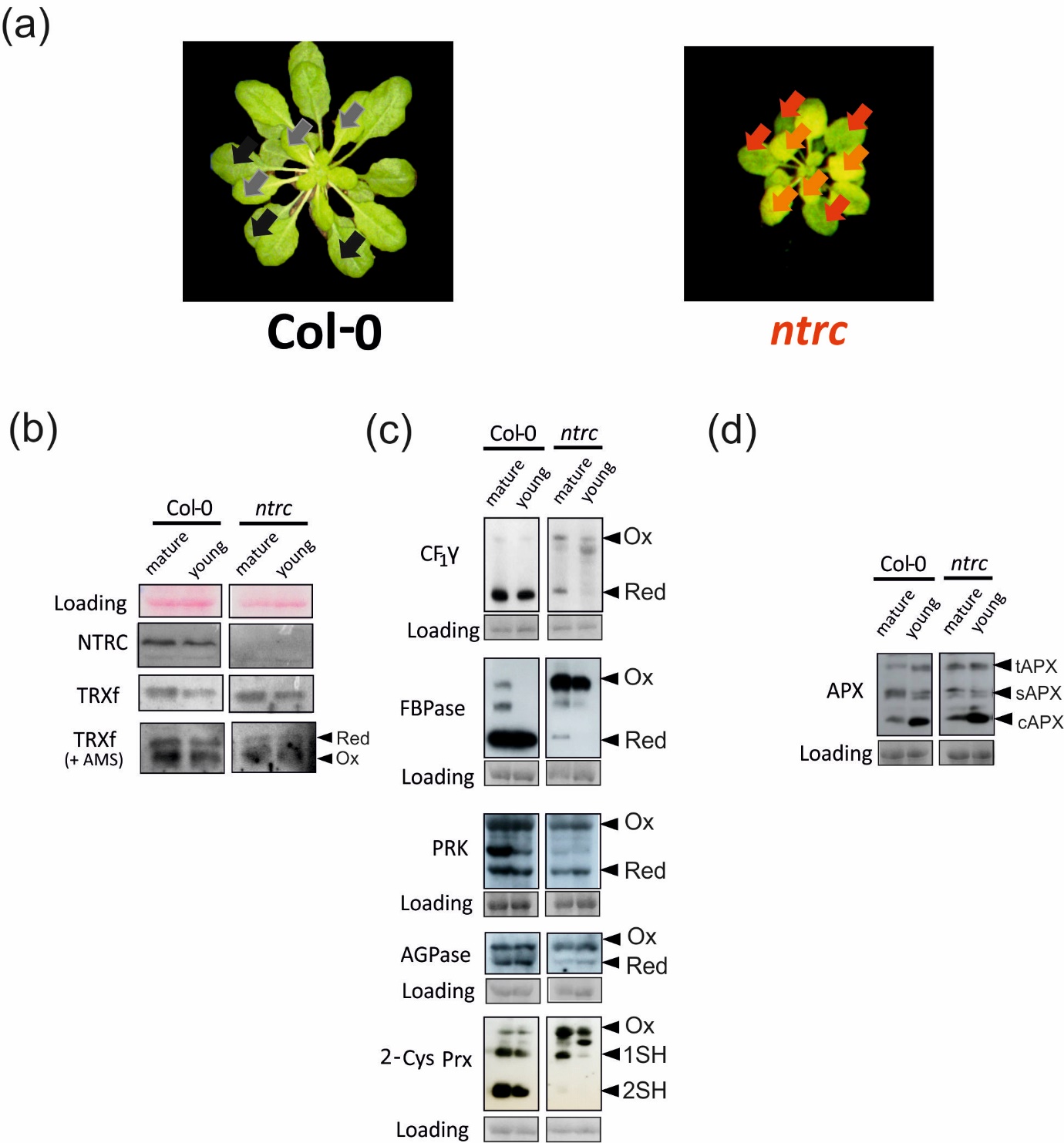
**

**Figure S6.** Redox states of chloroplast proteins in young and mature leaves of *Col-0* and *ntrc.*

(a) Young (grey and orange arrows) and mature (black and red arrows) leaves in 40 days old *Col-0* and *ntrc* plants used for estimation of redox states of chloroplast proteins. (b,c) The rosettes were illuminated at 200 μmol photons m^-2^ s^-1^ for 2 h before extraction of total protein from young and mature leaves of *Col-0* and *ntrc* in the presence of 10% trichloroacetic acid to preserve the thiol redox state of proteins. The proteins were labelled with 4-acetamido-4’-maleimidylstilbene-2,2’-disulfonic acid (AMS) (b) or methoxypolyethylene glycol maleimide MALPEG (c) as described earlier (Nikkanen *et al.,* 2016). Proteins were separated in SDS-PAGE before immunoblotting with antibodies raised against NTRC (Lepistö *et al.,* 2009), TRXf (Agrisera, AS14 2808), γ subunit of the ATP synthase (CF_1γ_) (Agrisera, AS08 312), fructose-1,6-bisphosphatase (FBPase) (kindly provided by Dr M. Sahrawy, CSIC, Spain), phosphoribulokinase (PRK) (Agrisera, AS07 257), ADP-glucose pyrophosphorylase (AGPase) (Agrisera, **AS11 1739)**, 2-cysteine-peroxiredoxins (2-Cys Prx) (kindly provided by Prof. F. J. Cejudo, Institute of Plant Biochemistry, University of Sevilla). Total amount of NTRC and TRXf as well as the redox state of TRXf are shown in the figure (b). Bands corresponding to oxidized (Ox) form of the proteins, partially reduced form of 2-Cys Prx (1SH), fully reduced form of 2-Cys Prx (2SH) (Nikkanen *et al.,* 2016) and fully reduced form of the proteins (Red) are marked in the figure (c). (d) The content of stromal (sAPX), thylakoid (tAPX) and cytosolic (cAPX) ascorbate peroxidases were determined by immunoblotting with APX antibody (Agrisera, AS08 368) recognizing all three APX isoforms in Arabidopsis. The immunoblots shown are representative of three independent experiments. Ponceau staining is used as loading control.

**REFERENCES**

**Kramer, D.M. and Crofts, A.R**. (1989) Activation of the chloroplast Atpase measured by the electrochromic change in leaves of intact plants. *Biochim. Biophys. Acta* **976**:28-41.

**Lepistö, A., Kangasjärvi, S., Luomala, E.M., Brader, G., Sipari, N., Keränen, M., Keinänen, M. and Rintamäki, E**. (2009) Chloroplast NADPH-thioredoxin reductase interacts with photoperiodic development in Arabidopsis. *Plant Physiol.* **149**, 1261-1276.

**Nikkanen, L., Toivola, J. and Rintamäki, E**. (2016) Crosstalk between chloroplast thioredoxin systems in regulation of photosynthesis. *Plant Cell Environ.* **39**, 1691-1705.
